# Analysis of Fecal Short-Chain Fatty Acids (SCFAs) in Healthy Children during the First Two Years of Life: An Observational Prospective Cohort Study

**DOI:** 10.3390/nu15020367

**Published:** 2023-01-11

**Authors:** Beata Łoniewska, Magda Fraszczyk-Tousty, Piotr Tousty, Karolina Skonieczna-Żydecka, Dominika Maciejewska-Markiewicz, Igor Łoniewski

**Affiliations:** 1 Department of Neonatology and Intensive Neonatal Care, Pomeranian Medical University in Szczecin, 70-111 Szczecin, Poland; 2 Department of Obstetrics and Gynecology, Pomeranian Medical University in Szczecin, 70-111 Szczecin, Poland; 3 Department of Biochemical Science, Pomeranian Medical University in Szczecin, 71-460 Szczecin, Poland; 4 Department of Human Nutrition and Metabolomics, Pomeranian Medical University in Szczecin, 71-460 Szczecin, Poland; 5 Sanprobi sp. z o.o. sp. k., 70-535 Szczecin, Poland

**Keywords:** short-chain fatty acids, newborn, children, perinatal factor, neonatology, gut microbiome, microbiota development

## Abstract

Short-chain fatty acids (SCFAs) are important metabolites of the gut microbiota. The aim is to analyze the influence of perinatal factors, which can affect the gut microbiota, on the concentrations of fecal SCFAs over the first two years of life. Gas chromatography was used to analyze SCFA in a total of 456 fecal samples from 86 children. Total SCFA concentrations increased until 12 months and stabilized after that. Antibiotic treatment during pregnancy was associated with an increase in acetic acid, propionic acid and total SCFA in meconium and a decrease in the same SCFAs at 6 months. Butyric acid was increased after Caesarean delivery until 1 month. In formula-fed children, propionic acid (at 1 month) and butyric acid and total SCFA (at 12 months) were increased. Acetic and linear butyric acids and total SCFAs were also increased at 12 months in children born vaginally that were also formula-fed. Higher butyric acid was observed in children of mothers with normal pre-pregnancy weight and adequate weight gain during pregnancy. Butyric acid was also elevated in 6-month-old infants with a higher body weight (≥85th percentile). Acetic acid concentrations were significantly higher in 2-year-old females vs. males. We conclude that perinatal factors are linked to changes in fecal SCFAs and further long-term epidemiological studies are warranted.

## 1. Introduction

After birth, the gut microbiota of the infant consists of mainly aerobes as well as facultative anaerobic bacteria and *Enterobacteriaceae*. The total number of bacterial cells is low, and so is the diversity. Within a few days of life, the gut environment becomes more anaerobic, and the *Bifidobacterium* abundance increases. This genus dominates in the first months of life. When solid foods are introduced into the diet, i.e., from about six months of age, the microbiota develops more rapidly. It begins to resemble an ecosystem typical of an adult human being. However, the process during which Firmicutes and Bacteroidetes start to dominate takes about 2–3 years of life after which these two phyla make up the majority of the human gut microbiome [1,2].

From an evolutionary point of view, the most important function of the bacteria in the gut is the metabolism of dietary compounds and other xenobiotics, including drugs. The bacterial enzyme apparatus helps to digest nutrients ingested by the host. Additionally, foods that are nondigestible for humans can be further decomposed by the gut microbiota [3]. As a result of the fermentation of complex carbohydrates and glycans, such as mucin or oligosaccharides present in human milk, short-chain fatty acids (SCFAs) are generated [4].

About 500–600 mmoles of SCFAs are produced daily, and this pool depends primarily on the amount of fiber consumed and the composition of the microbiome [5]. SCFA function as free acids and do not require protein binding [6]. After SCFA, mainly butyrate, are absorbed by intestinal epithelial cells [7], they enter the citric acid cycle to synthesize ATP [8]. SCFAs that are not used by intestinal epithelial cells pass through the portal vein to the liver where they are metabolized [8]. Acetate is an acid that is found in peripheral circulation. SCFA can cross the blood–brain barrier [9,10], acting as signaling molecules and transmitting information along the gut–brain axis. In addition, acetate serves as an energy source for the brain’s astrocytes [11]. The physiological functions of SCFA are elegantly discussed elsewhere [5,11,12]. Butyrate is produced mainly by bacteria of the genera: *Clostridium*, *Eubacterium* and *Fusobacterium* [13], but the most productive are *Clostridium leptum*, *Roseburia* spp., *Fecalibacterium prausnitzii* and *Coprococcus* spp. [14]. Propionate is a metabolite of Bacteroidetes and *Propionibacterium* [15]. It is recognized that Firmicutes can then metabolize the acetic acid produced by Bacteroidetes to produce butyric and propionic acids [16]. However, the synthesis pathway of individual SCFA is variable; butyric or propionic acids can be converted to acetic acid with the participation of *Acetobacterium*, *Acetogenium*, *Eubacterium* or *Clostridium* bacteria, or vice versa, when the number of butyrate-producing microorganisms increases [17].

These major bacterial metabolites are critical to the human gut. Acetate, while being transferred to peripheral tissues, might take part in lipogenesis and cholesterol metabolism, as well as acting as a satiety modulator. Butyrate is an energy source for colonocytes and has the ability to control gene expression through the inhibition of histone deacetylase. Importantly, it was demonstrated that acetic and butyric acids might play a role in bacterial gene expression as evidenced in *Salmonella* and *Escherichia*. Propionate can be utilized by many cells, including epithelial ones, and, once it enters the liver, is a substrate for gluconeogenesis [18].

In our previous study, we showed that several perinatal factors, including mode of delivery, antibiotic use, type of feeding and maternal body mass index, influence gut barrier integrity at 7 days of life as measured using calprotectin and zonulin [19]. However, a subsequent analysis of this cohort over two years of life demonstrated an elevation of zonulin levels up to 12 months and decreased calprotectin levels at 6 months [20]. In another study involving the same cohort, we observed that *Ruminococcus* (torques group) played a pivotal role in controlling paracellular permeability, whilst the abundance of *Staphylococcus*, *Staphylococcaceae*, *Ruminococcaceae* and *Clostridiales* may be linked to the development of the immune system. Nevertheless, causation was not proved [21].

Of note, in previous studies, we did not assess the fecal SCFA synthesis, which was a significant limitation. Therefore, in this study, we decided to analyze the SCFAs in fecal samples collected longitudinally over 24 months from 86 subjects. We aimed to test the hypothesis that perinatal factors, such as mode of delivery, type of feeding, exposure to antibiotics, bodyweight of mothers and children and sex, can influence the synthesis of SCFAs.

## 2. Materials and Methods

### 2.1. Patients

**Figure 1:**
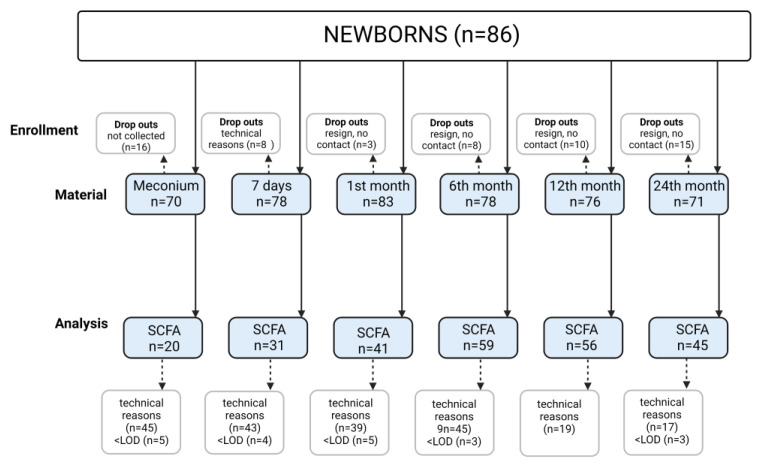
Study flow chart. Technical reasons—problems with collecting stools from diapers due to liquid consistency and small volume; LOD—limit of determination.

The present study is a continuation of assessments carried out on a cohort of mothers and their children who were patients of the Department of Obstetrics, Gynecology and Neonatology, the Pomeranian Medical University/Independent Public Clinical Hospital No.2 in Szczecin from March 2015 to April 2016. The population has been extensively described in previous publications [19,20,21]. In total, 86 newborns, from single pregnancies and born between 37 and 41 weeks of gestation, were included in the study (Figure 1). Samples (*n* = 456, in total) were taken at the following time points—meconium (P1, *n* = 70), 7 days (P2, *n* = 78), 1 month (P3, *n* = 83), 6 months (P4, *n* = 78), 12 months (P5, *n* = 76) and 24 months (P6, *n* = 71) after birth. The characteristics of the study group are presented in Table 1 and Table 2.

### 2.2. Brief Description of the Conducted Measurements

Data on maternal BMI, weight gain during pregnancy and antibiotic therapy during pregnancy were recorded from the Pregnancy Chart and the history collected from the mothers (objective data were not available because a universal system of medical data collection does not exist in Poland). The condition of each newborn after birth was assessed according to the Apgar scale (newborn screening tool used after birth describing a tolerance of the birthing process and providing information on how well the baby is doing outside the womb [22]) and on the basis of the results of umbilical-cord blood-gas assessments. Inclusion criteria were as follows: Apgar scale after 3 min of life > 7 points; umbilical cord blood pH > 7.2; and cord blood analysis showing: *C*-reactive protein (CRP) < 5 mg/L and Interleukin 6 (IL6) < 30 pg/mL (i.e., no clinical signs of congenital infection). The study excluded children born to mothers with autoimmune diseases (including type 1 diabetes), pregnancies complicated by gestational diabetes mellitus (GDM), with HELLP syndrome (Hemolytic anemia, Elevated Liver enzymes, Low Platelet count), prematurely born (before 37 weeks of gestation), in asphyxia (Apgar ≤ 7 after 3 min of age), with congenital infection (clinical signs of infection: increased CRP, IL6), or with birth defects as well as those not conforming to the inclusion criteria. Weight measurements referred to female and male growth charts created by the World Health Organization: Weight-for-age: Birth to 2 years (percentiles) (https://www.who.int/tools/child-growth-standards/standards/weight-for-age; accessed on 4 January 2023).

All newborns were solely breast-fed in the first week of life and discharged home. The breastfeeding period took into account exclusive and partial breastfeeding times. Formula feeding involved giving the baby formula milk; however, the composition of the milk formula was not specified due to the different formulas that are available on the market.

The first two samples and metadata collection took place in the hospital. At around 1, 6, 12 and 24 months old, the authors contacted the parents to perform interviews and obtain stool samples in order to determine the SCFA concentrations (max. deviation 3 days for 1 and 6 months and 7 days for 12 and 24 months). The interview included data on the health of the child (infections and antibiotic therapy), diet and weight. All measurements of the infant weights were referred to female and male growth charts created by the WHO (The WHO Child Growth Standards).

The children were healthy and did not take antibiotics at the time the samples were obtained.

### 2.3. Ethical Information

Consent to perform the study in the Department of Neonatal Diseases was obtained from the Bioethics Committee of the Pomeranian Medical University with Resolution No. KB-0012/55/14 of 30 June 2014. The study followed the Declaration of Helsinki (2013). Written informed consent was obtained from participating patients (parents).

### 2.4. Stool Sampling Scheme

The material for the research was stool collected from newborns and infants. Feces in an amount of at least 500 mg L were collected in a 2 mL Eppendorf Tubevial. The stool, in accordance with the established procedure, was collected from the diaper by previously trained parents, and subsequently stored in a home refrigerator (for a maximum of eight h) until it was collected by the researcher. Transport to the laboratory lasted up to 60 min at a temperature range from +6 to +8 °C, and then the material was stored at −20 °C until the analyses were performed.

### 2.5. Isolation of SCFA

A 0.5 g fecal sample was suspended in a tube containing 5 mL of water and mixed intensively for 5 min using laboratory vortex. Using 5 M HCl solution, the pH of the suspension was adjusted to 2–3. The samples were further shaken for 10 min and centrifuged (Eppendorf 5804, Darmstadt, Germany) for 20 min at 5000 rpm. The supernatant was filtered (Ø 400 µm) and transferred to a standard chromatographic vial for gas chromatography analysis.

### 2.6. Gas Chromatography

The following SCFA were analyzed as previously described [23]: acetic acid (C 2:0), propionic acid (C 3:0) and butyric acid (branched and linear) (C 4:0 *n*). Chromatographic analyses were carried out using the Agilent Technologies 7890 A GCsystem with a flame ionization detector (FID). Fused-silica capillary column with a free fatty acid phase (DB-FFAP, 30 m × 0.53 mm × 0.5 um) was used. The carrier gas was hydrogen at a flow rate equal to 14.4 mL/min. The initial temperature (100 °C) was maintained for 0.5 min, and then raised to 180 °C with ramping of 8 °C/min to be constant for 1 min. Subsequently, the temperature was increased to 200 °C (ramping 20 °C/min), to finally reach 200 °C and be sustained for 5 min. The injection volume was 1 μL and the run time of a single analysis was 17.5 min.

SCFAs were identified qualitatively and quantitatively. Qualitative analysis was performed by comparing the retention time of a certain molecule to the standard—2-ethyl butanoic acid. The concentrations of individual acids were converted against the internal standard. ChemStation Software (Agilent Technologies, Santa Clara, USA) was used for quantification.

### 2.7. Statistical Analysis

The results of the study were statistically analyzed similarly as in previous analyses published for the present cohort [19,20]. The normality of the distribution was checked with the help of the Kolmogorov–Smirnov test. Most of the data were not normally distributed; therefore, non-parametric tests were used. Mann–Whitney or Kruskal–Wallis (along with Dunn’s with Bonferroni correction for post hoc analysis) tests were used for unpaired data (comparisons between groups independently for each time point), and the Wilcoxon signed rank test was used for paired data (comparisons between time points independently for each group to asses dynamics of change). An analysis of variance (ANOVA) with repeated measures on rank-transformed total SCFAs was used to account for the use of antibiotics and the transition from breastfeeding. Either the conversion of feeding status or the use of antibiotics between pertinent time points served as the between-subject factor. If there was evidence of antibiotic use between the two time points, the antibiotic conversion was established. If the feeding status changed from natural to artificial between the two time points, the feeding conversion was determined. A Spearman test was used to analyse the correlation between the variables. The following formulas were used to calculate the effect size: for Mann–Whitney and Wilcoxon signed rank tests: r = (Z/$\sqrt{n}$), where Z = normalized U value and *n* = total number of observations; and for Kruskal–Wallis tests: epsilon-squared = $\left(\frac{H}{\left(n^{2}-1\right):\left(n+1\right)}\right)$, where H = H test statistic and *n* = total number of observations. For the multivariable analysis, we used multiple linear regression on rank-transformed SCFAs. Six models were fitted individually for every SCFA-dependent variable (all SCFAs, acetic acid, propionic acid, linear butyric acid, branched butyric acid and all butyric acids).Statistica ver. 13 software (StatSoft, Kraków, Poland) was used for the analysis. A 5% significance level was used.

## 3. Results

### 3.1. SCFA Concentrations over Time

**Figure 2:**
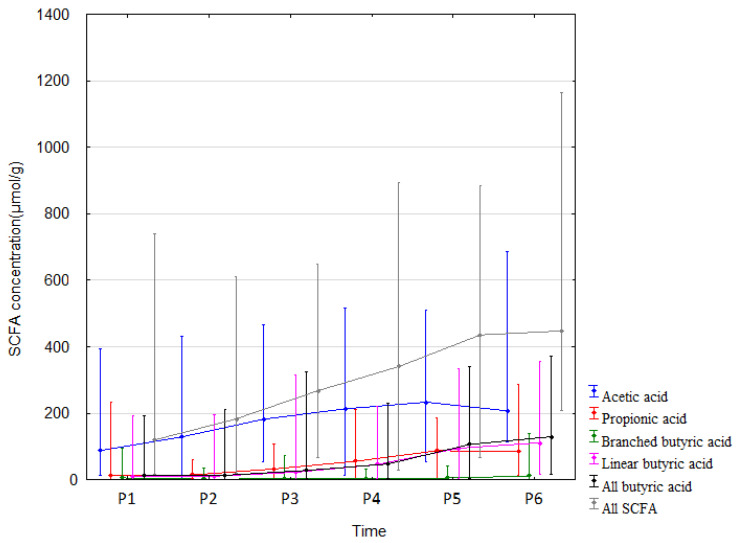
SCFA concentrations (median) over time (P1–P6 from left to right). Legend: Error bars represent range. From Wilcoxon paired test regarding time. Acetic acid: *p* < 0.05 P1 vs. P2, P4, P5, P6; *p* < 0.05 P2 vs. P3, P4, P5, P6. Propionic acid: *p* < 0.05 P1 vs. P2, P4, P5, P6; *p* < 0.05 P2 vs. P4, P5, P6; *p* < 0.05 P3 vs. P4, P5, P6; *p* < 0.05 P4 vs. P5, P6. Branched butric acid: *p* < 0.05 P2 vs. P5, P6; *p* < 0.05 P3 vs. P6; *p* < 0.05 P4 vs. P6; *p* < 0.05 P5 vs. P6. Linear butyric acid: *p* < 0.05 P1 vs. P4, P5, P6; *p* < 0.05 P2 vs. P4, P5, P6; *p* < 0.05 P3 vs. P4, P5, P6; *p* < 0.05 P4 vs. P5, P6. All butyric acid: *p* < 0.05 P1 vs. P4, P5, P6; *p* < 0.05 P2 vs. P4, P5, P6; *p* < 0.05 P3 vs. P4, P5, P6; *p* < 0.05 P4 vs. P5, P6. All SCFA: *p* < 0.05. P1 vs. P2, P4, P5, P6; *p* < 0.05 P2 vs. P3, P4, P5, P6; *p* < 0.05 P3 vs. P4, P5, P6; *p* < 0.05 P4 vs. P5, P6.

The measured SCFA concentrations in stool increased from the beginning of life (meconium sample) until 12 months of age. All SCFAs underwent similar changes, with the exception of acetic acid. Acetic acid concentration increased significantly only up to 1 month after birth and no significant increase was seen until the end of the study period (Table 3 and Figure 2). The measured concentrations are somehow similar to analyses by other authors [18]. Correlation analyses and effect sizes are shown in Supplementary Tables S1–S5. Given the significant changes in SCFAs over time (Figure 2), the impact of antibiotic and feeding changes was examined for the time points P3 vs. P4, P5, P6, and P4 vs. P5, P6. The use of antibiotics in children and changes in feeding had no impact on how SCFA concentrations changed over time (antibiotics: P3 vs. P4: F1, 31 = 0.73, *p* = 0.399, P3 vs. P5: F1, 28 = 0.59, *p* = 0.449, P3 vs. P6: F1, 22 = 0.024, *p* = 0.877, P4 vs. P5: F1, 43 = 0.05, *p* = 0.816, P4 vs. P6: F1, 36 = 1.01, *p* = 0.322; feeding: P3 vs. P4: F1, 30 = 2.26, *p* = 0.144, P3 vs. P5: F1, 27 = 0.003, *p* = 0.974, P3 vs. P6: F1, 21 = 0.57, *p* = 0.458, P4 vs. P5: F1, 42 = 0.72, *p* = 0.402, P4 vs. P6: F1, 35 = 0.10, *p* = 0.753).

### 3.2. Effect of Antibiotic Therapy during Pregnancy 

**Figure 3:**
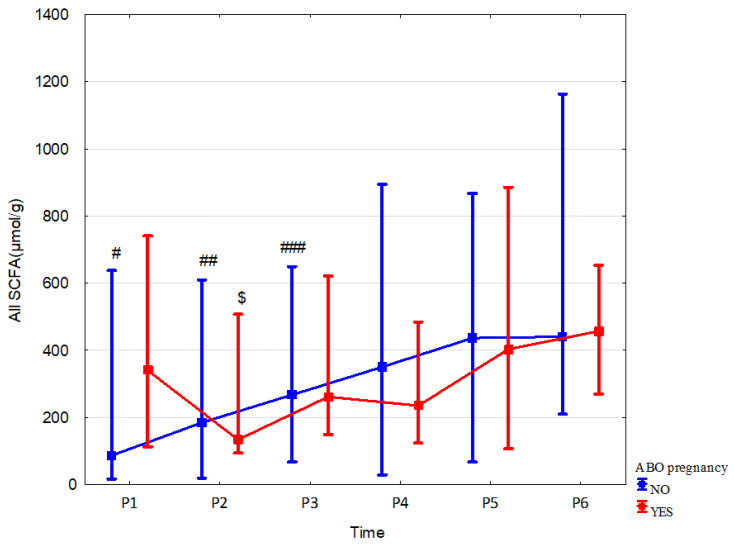
All SCFA concentrations (median) in children over time of mothers with (red line) or without (blue line) antibiotic therapy during pregnancy (ABO). Legend: Error bars represent range. Wilcoxon paired tests regarding time: (# No ABO, $ ABO): # *p* < 0.05 P1 vs. P2, P3, P4, P5 and P6; ##, $: *p* < 0.05 P2 vs. P3, P4, P5 and P6; ### *p* < 0.05 P3 vs. P4, P5 and P6.

Children of mothers who received antibiotics during pregnancy showed higher concentrations of acetic acid, propionic acid and total SCFAs in meconium (by 221.5% (*p* = 0.02, r = 0.51), 496.1% (*p* = 0.006, r = 0.58) and 291.6% (*p* = 0.026, r = 0.49), respectively) and a decrease in SCFAs mentioned above at 6 months (by 25.2% (*p* = 0.04, r = −0.26), 41% (*p* = 0.007, r = −0.34) and 32.9% (*p* = 0.019, r = −0.03), respectively) when compared to children not exposed to antibiotics in utero (Table 4, and Supplementary Tables S6 and S7). The greater dynamics of changes (measured over time for the same group—Figure 3 and Supplementary Figures S1 and S2) in the concentrations of all fatty acids and propionic acid in the stool were observed up to 6 months of age in the children of mothers who were not treated with an antibiotic. The dynamics of changes in acetic acid concentrations were similar in both groups; however, due to the small sample size in the group of the children of mothers treated with antibiotics during pregnancy, the observed changes were statistically insignificant. The antibiotic therapy of mothers during delivery and combined data on the effect of antibiotics administered during pregnancy or delivery and during pregnancy or delivery or childhood all combined did not significantly affect the total SCFA concentrations. It is important to note that a very small number of children were not exposed to antibiotics via the mother or during childhood, which likely affected the outcome of the statistical analysis. Other data and effect sizes concerning antibiotic therapy are shown in Table 4, Figure 3, Supplementary Tables S8–S12 and Supplementary Figures S1 and S2.

### 3.3. Effect of Delivery Mode 

**Figure 4:**
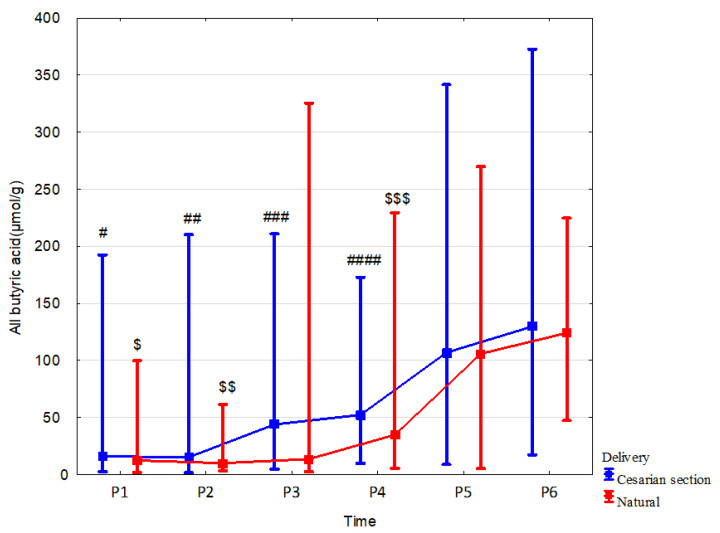
All butyric acid concentrations (median) in children over time according to delivery type: Cesarean section (blue line) or vaginal birth (red line). Legend: Error bars represent range. Wilcoxon paired tests regarding time, # (Cesarean section), $ (Vaginal delivery): #, $: *p* < 0.05 P1 vs. P5 and P6; ##, $$: *p* < 0.05 P2 vs. P4, P5 and P6; ### *p* < 0.05 P3 vs. P5 and P6; ####, $$$: *p* < 0.05 P4 vs. P5 and P6.

In children born via Cesarean section vs. born vaginally, fecal concentration of branched butyric acid was higher at 7 days by 72.9% (*p* = 0.049, r = 0.35), at 1 month linear butyric acid and total butyric acid were increased by 346.3% (*p* = 0.02, r = 0.36) and 225.2% (*p* = 0.037, r = 0.32), respectively. We also observed a tendency to increase the concentration of propionic acid at 1 month in children born vaginally by 52.5% (*p* = 0.066; r = –0.25). The mode of delivery did not affect the dynamics of changes in all fecal SCFA concentrations over time. Data and effect sizes are described in Table 5, Figure 4, Supplementary Tables S13–S15, and Supplementary Figures S3 and S4.

### 3.4. Effect of Feeding 

**Figure 5:**
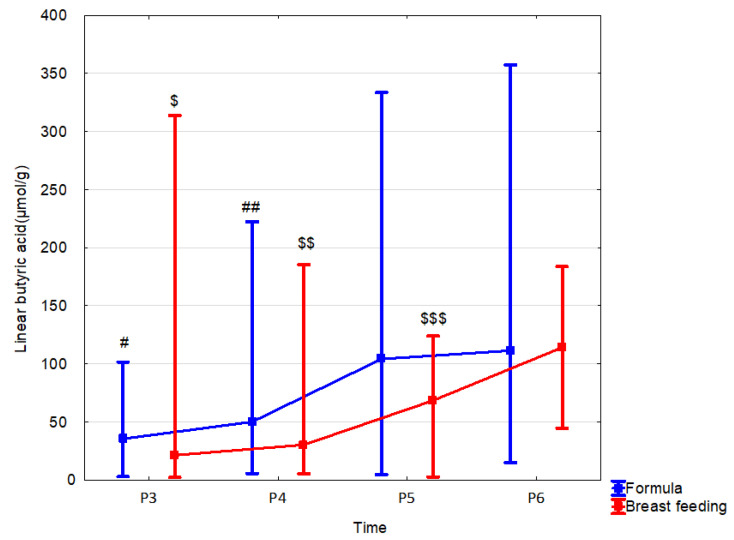
Linear butyric acid (median) in children over time according to the type of feeding: formula (blue line) or breast feeding (red line). Legend: Error bars represent range. Wilcoxon paired tests regarding time, # (formula), $ (breast feeding): #, $: *p* < 0.05 P3 vs. P5 and P6; ##, $$: *p* < 0.05 P4 vs. P5 and P6; $$$ *p* < 0.05 P5 vs. P6.

In formula-fed children, fecal concentrations of SCFAs were increased in comparison to those breast-fed as follows: propionic acid at 1 month by 106.0% (*p* = 0.059; r = −0.3); at 12 months: butyric acid linear and total by 52.3% (*p* = 0.049, r = −0.27) and 57.4% (*p* = 0.052, r = −0.26), respectively; total SCFA by 29.1% (*p* = 0.055, r = −0.26). Less changes in linear butyric acid levels were observed in breast-fed children, but these changes occurred throughout the whole observation period (Table 6, Figure 5 and Supplementary Table S16). The mode of delivery significantly affected the stool SCFA content depending on the type of feeding. In the group of formula-fed children that were born vaginally, increased content in the stool was observed at 12 months of age: acetic acid by 63.1% (*p* = 0.03, r = 0.5), linear butyric acid by 277.8% (*p* = 0.008, r = 0.61) and total SCFAs by 70.8% (*p* = 0.014, r = 0.56). In children born by *C*-section, no effect of feeding on stool SCFA concentrations was observed (Supplementary Tables S17 and S18).

### 3.5. Effect of Weight 

**Figure 6:**
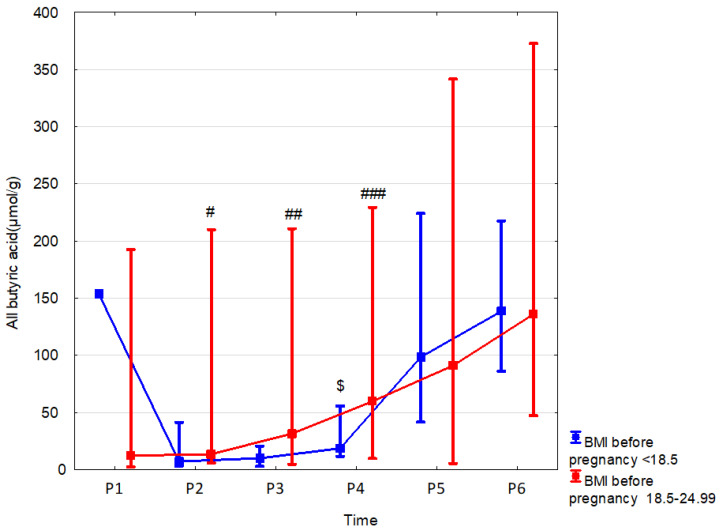
All butyric acid (median) in children over time according to the effects of BMI before pregnancy: BMI before pregnancy 18.5–24.99 (red line) or BMI before pregnancy < 18.5 (blue line). Legend: Error bars represent range. Wilcoxon paired tests regarding time, # (BMI before pregnancy 18.5–24.99), $ (BMI before pregnancy < 18.5): # *p* < 0.05 P2 vs. P5 and P6; ## *p* < 0.05 P3 vs. P6; ###, $: *p* < 0.05 P4 vs. P5 and P6.

In present study, we assessed the influence of the following parameters related to body weight on the SCFA content: 1/maternal BMI before pregnancy, 2/maternal weight gain during pregnancy, and 3/child body weight. We observed that, in the children of women with normal BMI (18.5 < 25) before pregnancy compared to those with underweight BMI (<18.8), the fecal linear butyric acid was higher by 75.3% (*p* = 0.023, r = 0.55) at 7 days and by 269.7% (*p* = 0.014, r = 0.04) at 6 months, and total butyric acid was higher by 243.9% (*p* = 0.002, r = 0.48) at 6 months. Linear butyric acid and butyric acid together were also greater at 6 months of age in children of women with normal BMI compared to the children of overweight women by 206.7% (*p* = 0.004, r = 0.44) and 243.9% (*p* = 0.002, r = 0.48), respectively (Table 7, Figure 6 and Supplementary Tables S19 and S20; Supplementary Figure S5).

**Figure 7:**
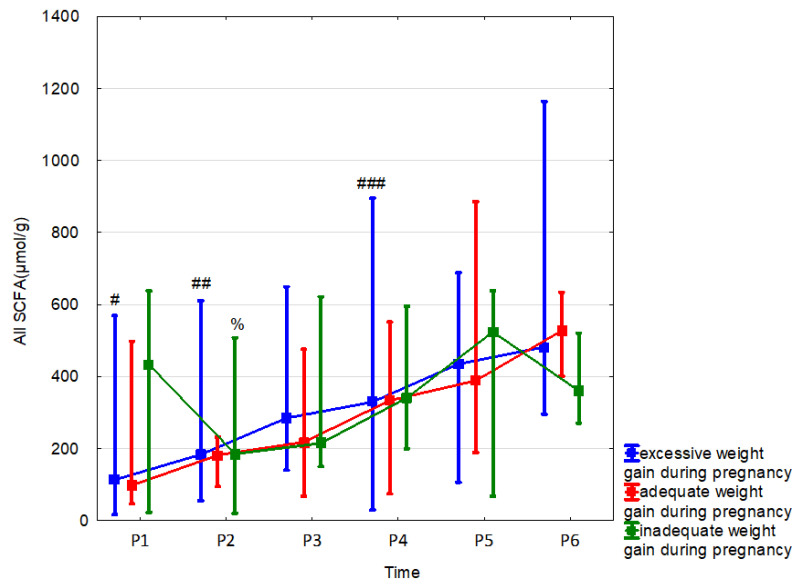
All SCFAs (median) in children over time according to the effects of weight gain during pregnancy. Excessive (blue line), adequate (red line) or inadequate (green line). Legend: Error bars represent range. Wilcoxon paired tests regarding time, # (excessive), % (inadequate): # *p* < 0.05 P1 vs. P3, P4, P5 and P6; ##, %: *p* < 0.05 P2 vs. P3, P4, P5 and P6; ### *p* < 0.05 P4 vs. P5.

Weight gain in pregnancy affected the concentration of butyric acid and the sum of fatty acids up to the 24th month of life (Table 8, Figure 7 and Supplementary Tables S21–S23; Supplementary Figures S6 and S7).

When looking at the effects of the weight of the child, several differences to note were observed. Subjects were classified in three categories: ≥85th percentile, >15–<85th percentile and ≤15th percentile. When comparing children in the ≥ 85th percentile vs. the > 15–<85th percentile, there was an increase in linear butyric acid and total butyric acid (78.7% (*p* = 0.37, r = 0.28) and 56% (*p* = 0.047, r = 0.26), respectively) at 6 months (Supplementary Table S24). No significant differences were observed in the fecal SCFA concentrations between children in the ≤ 15th percentile when compared to the ≥85th percentile (Supplementary Table S25).

### 3.6. Effects of Sex

**Figure 8:**
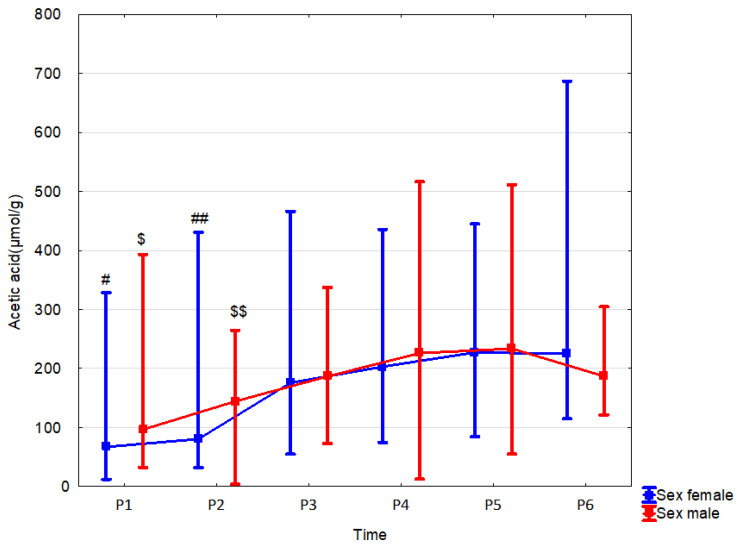
Acetic acid (median) in children over time according to the effect of sex: male (red line) or female (blue line). Legend: Error bars represent range. Wilcoxon paired tests regarding time, # (female), $ (male): #, $: *p* < 0.05 P1 vs. P4, P5 and P6; ##, $$: *p* < 0.05 P2 vs. P4,P5 and P6.

In female newborns, fecal acetate concentration at 12 months was significantly higher (by 20.3% (*p* = 0.039, r = 0.31)) than in males. Sex did not affect the dynamics of changes in SCFA concentrations over time (Table 9, Figure 8 and Supplementary Table S26).

### 3.7. Multivariable Analysis

We also performed a multivariable analysis in the form of a multiple linear regression on the rank-transformed response—short-chain fatty acids (SCFAs), accounting for all factor variables presented in Table 4, Table 5, Table 6, Table 7, Table 8 and Table 9: ABO (antibiotic therapy during pregnancy), delivery mode, feeding type, BMI before pregnancy, weight gain and sex. The findings of the univariable analysis (Table 4, Table 5, Table 6, Table 7, Table 8 and Table 9) and the multivariable analysis are merged and shown in Table 10.

The entries in the table show the time points at which a statistically significant associations was discovered to exist between the factor predictor and the response (SCFA). A univariate analysis is indicated by the U subscript, whereas a multivariable analysis is indicated by the M subscript. A UM subscript is used if both types of analyses found a significant association.

Both the univariable and the multivariable techniques have some inconsistencies in their findings. However, delivery mode at P2 (branched butyric), feeding type at P5 (linear butyric), BMI before pregnancy at P4 (linear butyric, all butyric) and weight gain at P6 (all SCFAs, linear butyric, branched butyric and all butyric) were all identified as significant predictors in both analyses (Table 10), implying that these factors may influence SCFAs independent of other factors.

## 4. Discussion

The content of SCFAs in the stool of an individual is an overall net measurement resulting from the production of these compounds by the intestinal bacteria, and their absorption, utilization and degradation in the gastrointestinal tract. The SCFA pool in feces reflects their content indirectly in the body, which cannot be measured in the blood of healthy newborns due to several ethical considerations.

This paper is a longitudinal study aimed at analyzing SCFAs in fecal samples collected over the first two years of life and the influence of different maternal and child parameters on the content of these molecules in feces. We demonstrates that the overall SCFA concentrations in stool increased until 12 months of age, except for acetic acid. A similar observation was made in other studies [25]. Acetate content increased significantly only until one month of life, which was also previously shown [26]. Increasing with the growth of children, absorption in the intestine (anatomically and functionally determined) and the in situ metabolism of SCFAs may be responsible for the earlier stabilization of SCFA excretion, despite the increased production of these compounds by the microbiota.

Variations in SCFA content reflect changes in the composition and function of the microbiota. Acetic acid is a primary SCFA [27,28] and is produced by most anaerobes, such as *Bacteroides* spp. and *Akkermansia muciniphila,* which also produce propionate [28,29]. *Clostridioides* also synthesizes butyrate, as do *Anaerostipes, Clostridioides, Coprococcus, Dorea, Eubacterium, Fecalibacterium, Roseburia* and *Ruminococcus* [30,31,32]. Additionally, in a cross-feeding process, *Eubacterium* and *Anaerostipes* genera convert acetic acid into butyrate with the help of *Bifidobacterium* [33]. In previous research conducted involving the children belonging to the present study cohort, dynamic changes in the composition and metabolic potential of the microbiota, including the prediction of the production of SCFA, were observed in the first two years of life [21]. We confirmed in this study that the composition of SCFAs in the stool of children aged 1 and 2 years is similar to the values observed in adults, which indicates the essential role of the first years of life in the development of the metabolic function of the microbiota. This observation underlines that this period is critical to evaluate factors mediating certain bacteria abundance and their metabolism.

Various perinatal factors influenced both the SCFA content in the stool at particular time points and the dynamics of SCFA content during this study. We here confirmed using the univariable and the multivariable analysis that feeding type at 12 months (reg. linear butyric), delivery mode at 7 days (reg. branched butyric), BMI before pregnancy at 6 months (reg. linear butyric, all butyric) and weight gain at 24 months (all SCFAs, linear butyric, branched butyric and all butyric) are factors significantly modulating SCFA synthesis.

One of the crucial factors was diet. In non-breast-fed children, higher concentrations of propionic acid in the stool at one month and acetic and butyric acid at 12 months of age were measured. Interestingly, the differences in SCFA levels at time points were observed during the entire study period in breast-fed children. In formula-fed ones, SCFA excretion stabilized at 12 months of age. In the multivariable analysis, this factor was, however, only significant regarding linear butyrate at 12 months. These observations are partially confirmed by the results of a recently published meta-analysis from our group [18] and might result from a difference in microbiota composition between breast- and formula-fed children [34,35,36,37,38,39]. Additionally, a meta-analysis of 1825 stool samples from 684 infants found that microbiome diversity, maturity, the relative abundance of Bacteroidetes and Firmicutes and the predicted pathways of carbohydrate metabolism were greater in formula-fed infants [40]. Similarly, artificially fed infants showed an increase in the relative abundance of Bacteroidetes and Firmicutes and bacteria at low taxonomic levels compared to their breast-fed counterparts [41]. Of note, bacterial diversity in infants was not dependent on the type of diet [42,43,44]. Koenig et al. found that the introduction of complementary foods also increased the concentration of SCFAs [28]. Differding et al. [45] proved that the early, not later, introduction of such foods alters gut microbiota composition and elevation of butyric acid concentrations in stool up to 1 year of age. In particular, the blooming of *Bilophila wadsworthia* and *Lachnospiraceae Roseburia* is associated with elevated butyrate content at 3 and 12 months of life. *L. Roseburia* is a well-known butyrate producer with a proven impact on gut mucosa [32] and cardiometabolic health [46]. In the first few months of life, breastfeeding may promote SCFA production. However, we did not observe that in our study. Brink et al. demonstrated higher butyric acid content in the stools of breast-fed infants compared to formula-fed infants [47], but others [48] proved that within the SCFA pool, only lactate was higher at the age of 3–5 months. Additionally, the relative content of acetate was higher in the exclusively breast-fed children and independent of type of delivery, intake of antibiotics and other factors. Such a high proportion of acetate was confirmed in 4-week-old babies naturally fed [49].

Human milk contains molecules positively affecting gut microbiota and, therefore, SCFA synthesis. Human milk oligosaccharides (HMO)—prebiotics for gut bacteria—not present in formula—might be the reason for the elevated acetate content in breast-fed infants [50], especially as the close linkage between HMO, mother’s milk and infant gut ecosystem has been established [51,52,53,54]. Additionally, many other women’s milk components affect gut bacteria [55,56]. These are maternal immunoglobulins [57], cytokines and growth factors, defensins and cathelicidins [58], lysozyme, amino acids and others, all of which might stimulate immunity [59]. In addition, an important human biomolecule, lactoferrin, impacts bacteria in the gut by limiting the uptake of iron ions [60]. It follows then that breastfeeding may lead to mild but continuous changes in the microbiota in the first two years of life, and artificial feeding causes its faster maturation. However, one must note that increased fecal SCFA in formula-fed infants might negatively alter host metabolism [61]; however, this might only be the association not causation. 

The impact of mode of delivery on fecal SCFAs is not clear-cut. We here demonstrated that, in children born via Cesarean section (*C*-section), the fecal concentration of butyric acid was higher until one month of life, but propionic acid concentration tended to be higher at one month. In multivariate analyses, we found that delivery mode significantly affected branched butyric concentration at seven days. The negative impacts of *C*-section on gut microbiota—lower counts of *Bifidobacterium*, *Bacteroides* and *Lactobacillus* and diminished bacterial diversity—compared to vaginal birth have already been confirmed [62,63]; however, a short improvement in biodiversity metrics introduced by *C*-section has also been demonstrated [64]. In another study, the impact of birth mode on microbiota lasted only a few days [65]. Mueller et al. [66] found that *C*-sections might favor potential pathobionts blooms at 3 and 12 months, along with high butyrate content in the stool. The results thus indicate that bacteria (especially *Lachnoclostridium*) that are enriched in the gut of children born via *C*-section produce butyrate, not all other SCFAs, to a large extent [67]. Delivery by *C*-section may reduce the absorption of butyrate through the apical membrane of the colonocytes, resulting in an excessive excretion of butyrate in the stool. Because higher stool butyrate levels may be associated with excessive weight gain, as shown in mice [68] and with obesity [69] and poor cardiometabolic health in men [70], this can explain the adverse health effects of *C*-section birth. Further research is needed to confirm this relationship.

The effect of birth mode on the stool SCFA concentrations observed by us may be related to the complexity of factors influencing microbiota metabolism [71]. An interesting observation is the lack of differences in SCFA concentrations in the group of children born through *C*-section when comparing type of feeding. It suggests that *C*-section birth eliminates the beneficial influence of breastfeeding on the metabolic activity of the microbiota, although Wu et al. observed that breastfeeding might stabilize SCFA metabolism in *C*-section-born infants [71]. The influence of the indications for *C*-sections on the microbiota status of newborns should also be taken into account, especially since the percentage of *C*-sections in the cohort observed here exceeded 60%.

An interesting observation was the increased content of acetic acid, propionic acid and total SCFAs in the meconium of children of women who took antibiotics during pregnancy. Meconium is the first stool of a newborn baby and is usually passed for the first 1–5 h after birth [72]. It begins to form in the fetal digestive tract between 12 and 16 weeks of pregnancy and continues until birth [73]. Meconium consists of debris of amniotic fluid, exfoliated skin and gastrointestinal cells, gastrointestinal secretions and enzymes, lanugo, fatty material from vernix caseosa, cholesterol and sterol precursors, bile acids and salts, blood group mucopolysaccharides [74] and proteins [75], sugars [76] and trace elements [77]. There are very few studies on SCFAs in meconium [26,78]. SCFAs, produced by various anaerobes and via different fermentation pathways, are formed mainly in the cecum and the ascending part of the colon [27]. The primary source of SCFAs produced by microorganisms is the unabsorbed portion of the food that reaches the large intestine. The amounts of SCFAs digested and secreted are presumably negligible under normal conditions [79]. The presence of bacterial DNA in the amniotic fluid [80] suggests that the activity of the intestinal microbiome and compounds derived from microbial metabolism may play a role in the development of the fetus in a later period. Additionally, transiently colonizing pregnant female mice showed that the maternal microbiota shapes the offspring’s immune system [81] (without colonization of the digestive tract), suggesting the role of bacterial metabolites in this process. For example, in piglets, meconium contains SCFAs (especially acetate and propionate), suggesting that the action of the microorganisms found in the intestine begins before birth [82]. Additionally, SCFAs can also reach the fetus through the placenta as low levels are found in the blood of the mother [83,84]. We previously showed that antibiotic therapy during pregnancy might result in increased calprotectin in the stool on the seventh day of life [20,21], which is associated with the modulation of the immune system and affects the intestinal microbiota [85]. The mechanism behind this observation is unknown and requires further investigation, especially as, at 6 and 24 months of age, SCFA concentration in the stool of children of women taking antibiotics during pregnancy is reduced, and the stabilization of propionic acid excretion occurs later. Pregnant women are generally treated with ampicillin, which crosses the placenta and can therefore directly affect the microbiota in the fetus [86]. It also crosses into the milk, but this did not affect the SCFA excretion in this study. Due to the small size of the study groups and inconsistencies arising from the results of the multivariable analysis, further research on the effect of prophylactic antibiotic therapy during childbirth and in children on SCFA synthesis is needed. The obtained results suggest caution when using antibiotics during pregnancy.

We also analyzed the relationships between the body weight of mothers and children with SCFA composition in the stools of newborns. Reduced and increased BMI before pregnancy was associated with increases in butyric acid content in children stools at six months of age. Greater weight gain in pregnancy affected the concentration of butyric acid and the sum of fatty acids in children at 24 months of life. Child weight in the ≤15th percentile was associated with an increased concentration of butyric acid at six months of age. In the multivariate analysis, BMI before pregnancy was significantly affecting butyrate at 6 months. Regarding weight gain all SCFAs, linear butyric, branched butyric and all butyric were affected by this variable but at the 24 month only. Studies on the relationship between SCFAs and body composition have shown obese adults to have higher levels of SCFAs in stool samples than lean individuals [69,87,88]. Plasma butyrate, propionate and acetate concentrations have a stronger positive relationship with the body fat composition compared to the Firmicutes/Bacteroidetes ratio in children 9 to 18 years of age [89]. These results suggest that the gut microbiota that produce SCFAs may be able to absorb more energy from the diet and, in some cases, lead to the development of a higher BMI. However, not all studies support an association between SCFA-producing bacteria and weight gain, and their anti-obesity effects have also been reported [90,91]. For example, acetate may reduce appetite and the amount of body fat and improve glucose tolerance [92,93]. In addition, SCFA dietary supplementation led to weight loss and improvement in insulin sensitivity in mice without changing eating patterns or their degree of physical activity [94]. The application of propionate to the colon of obese people resulted in a significant reduction in appetite, weight and obesity, as well as improved insulin sensitivity [95,96]. These findings suggest a more complex relationship between the gut microbiota, SCFAs and obesity than simply increasing the energy obtained from the diet.

Our study had some limitations. First, we did not evaluate the microbiota composition, which could shed more light on the mechanism of the observed changes. However, our previous research confirms both the dynamics of microbiota changes and changes in intestinal permeability and immune functions during this period of life [19,20,21]. Another limitation of our study was that only the analysis of SCFA was performed in stool samples. The concentration of metabolites in the feces is a function of the production, absorption, use by other microorganisms and transit time of the stool. It is estimated that 95% of SCFAs produced in the intestine are rapidly absorbed, and only 5% is excreted in the feces [94]. However, it was not possible to accurately analyze SCFAs in the blood of healthy newborns and small children for ethical reasons. Therefore, at present, only the analysis of stool samples is a non-invasive and rational alternative method of assessing SCFAs for epidemiological cohort studies. The limitations of the study also include the large number of samples that could not be analyzed due to technical reasons (mainly, too small amounts of material). Obtaining material from neonates and young children, especially over a two-year period, is associated with various technical and organizational difficulties. Our study was post hoc and SCFA analysis was performed as the last measurement; thus, we ran out of sample in several cases, especially because several analyses were already conducted on the same set of samples [19,20,21]. The amount of material analyzed guaranteed the correct result, which were verified in accordance with the established analytical methodology [23]. We did not perform a formal sample size analysis as the cohort had already been defined and the present study was of post hoc type. We, however, analyzed the effect size, and the obtained results usually had average effects. In addition, the size of our cohort was comparable to the number of children studied in other clinical trials dealing with similar topics [18]. At last, due to number of milk formulas present in the market, such information was not provided. Overall, our study did not present information on whether any pro- and prebiotics were ingested by the infants.

## 5. Conclusions

The obtained results indicate dynamic changes in the concentration of SCFAs in the stool in children in the first year of life, followed by the stabilization of their concentration in the stool. In addition, the examined maternal and fetal factors, such as antibiotic intake during pregnancy, feeding method, mode of delivery, pre-pregnancy body weight, changes in pregnancy body weight and the weight and sex of the children, affected SCFA excretion in the stool in various ways. Unfortunately, the conducted study did not allow us to determine the long-term health consequences of these observations. However, it can be speculated that breastfeeding and proper weight control in mothers and children have beneficial effects on health by influencing the composition of SCFAs in the stool and, therefore, the development of the gut microbiota. In contrast, antibiotic therapy during pregnancy and *C*-section may have an unfavorable effect. However, these speculations must be approached with great care, and long-term epidemiological studies are warranted to further determine the mechanism of these changes.

## Figures and Tables

**Table 1:** Group characteristics: newborns and mothers.

Characteristic	Newborns (*N* = 86)			
Gender: (% male)	54%			
Birth weight (g)				
(mean ± SD)	3441 ± 463			
(range)	2140–4960			
≤15th percentile	15% (*n* = 13)			
≥85th percentile	15% (*n* = 13)			
Characteristic	Mothers (*N* = 86)			
Vaginal childbirth	37% (*n* = 32)			
Antibiotic therapy during pregnancy	30% (*n* = 26)			
Antibiotic therapy at delivery	81% (*n* = 69)			
BMI before pregnancy (%)	<18.5	18.5 < 25	25 < 30	>30
	13.2%	53.0%	20.5%	13.3%
BMI before delivery (%)	<18.5	18.5 < 25	25 < 30	>30
	0%	16.9%	33.7%	49.4%
Gestational weight gain	inadequate	adequate	excessive	
	22.8%	17.7%	59.5%	

**Table 2:** Group characteristics: children up to 24 months old.

Characteristic	Age of Children (Months)			
	1	6	12	24
	*n* = 83	*n* = 78	*n* = 76	*n* = 71
Gender (% male)	49.3% (*n* = 41)	48.7% (*n* = 38)	47.3% (*n* = 36)	43.7% (*n* = 31)
Method of delivery (% vaginal)	33.7% (*n* = 28)	34.6% (*n* = 27)	34.2% (*n* = 26)	32.4% (*n* = 23)
Antibiotics (%)	2.53% (*n* = 2)	21.25% (*n* = 17)	40.8% (*n* = 31)	74.6% (*n* = 53)
Birth weight:				
≤15th percentile	14.5% (*n* = 12)	12.8% (*n* = 10)	13.2% (*n* = 10)	12.7% (*n* = 9)
≥85th percentile	14.5% (*n* = 12)	14.1% (*n* = 11)	13.2% (*n* = 10)	12.7% (*n* = 9)
Mass (kg) at each age:				
(mean ± SD)	4.49 ± 0.612	7.907 ± 1.099	10.07 ± 1.08	12.89 ± 1.684
(range)	(2.780, 5.970)	(6, 10)	(7.89, 12.5)	(10, 17)
≤15th percentile *	12.0% (*n* = 10)	21.8% (*n* = 17)	1.3% (*n* = 1)	2.8% (*n* = 2)
>15–<85th percentile *	63.9% (*n* = 53)	53.8% (*n* = 42)	65.8% (*n* = 50)	62% (*n* = 44)
≥85th percentile *	24.1% (*n* = 20)	24.4% (*n* = 19)	32.9% (*n* = 25)	35.2% (*n* = 25)
Feeding method (% non-breast-fed)	17.1% (*n* = 14)	51.2% (*n* = 40)	85.4% (*n* = 65)	95.7% (*n* = 68)

* classification was carried out at each time point of the sampling.

**Table 3:** Concentrations of SCFAs in children at six study time points.

Stage	Meconium (P1) *n* = 20	7 Days (P2) *n* = 31	1 Month (P3) *n* = 41	6 Months (P4) *n* = 59	12 Months (P5) *n* = 56	24 Months (P6) *n* = 45
Acetic acid (µmol/g) median (range)	88.49 (12.42–393.51)	129.26 (4.11–430.51)	182.21 (55.12–466.02)	213.1 (12.55–516.45)	233.49 (55.12–511.2)	207.5 (114.7–686.93)
Propionic acid (µmol/g) median (range)	13.93 (1.57–234.50)	14.71 (4.76–61.74)	32.87 (5.07–106.6)	55.69 (3.16–310.69)	87.48 (2.60–187.6)	86.36 (14.58–285.45)
Branched butyric acid (µmol/g) median (range)	5.81 (0.07–94.75)	1.95 (0.087–35.71)	3.24 (0.18–72.18)	3.25 (0.07–31.6)	5.52 (0.59–4.23)	11.3 (1.26–140.2)
Linear butyric acid (µmol/g) median (range)	8.96 (1.19–191.3)	11 (1.47–195.5)	22.95 (2.18–313.87)	45.9 (5.34–222.33)	95.19 (2.54–333.58)	111.34 (14.9–357.45)
All butyric acid (µmol/g) median (range)	14 (1.7–192.5)	13.6 (1.61–210.1)	28.39 (2.37–325.46)	47.7 (5.42–229.39)	106.33 (5.14–341.71)	129.72 (17.17–372.79)
All SCFA (µmol/g) median (range)	119.95 (16.5–740.77)	183.21 (19.44–610.01)	267.57 (67.32–649.06)	340.54 (29.27–894.84)	435.65 (67.32–885.64)	447.63 (209.65–1163.73)

**Table 4:** Effects of antibiotic therapy during pregnancy on all SCFA concentrations.

Stage	Meconium (P1)	7 Days (P2)	1 Month (P3)	6 Months (P4)	12 Months (P5)	24 Months (P6)
ABO YES	*n* = 6	*n* = 8	*n* = 10	*n* = 13	*n* = 17	*n* = 9
All SCFA (µmol/g) media n (range)	340.09 (113.09–740.77)	133.73 (94.15–507.52)	261.69 (149–621.41)	235.14 (123.94–484.31)	403.65 (106.55–885.64)	456.73 (270.29–653.56)
ABO NO	*n* = 14	*n* = 23	*n* = 31	*n* = 46	*n* = 39	*n* = 36
All SCFA (µmol/g) median (range)	86.85 (16.5–637.63)	184.78 (19.64–610.01)	267.57 (67.32–649.06)	350.51 (29.27–894.84)	436.6 (67.32–867.37)	440.52 (209.65–1163.73)
*p*, r	0.026 (0.49)	0.55 (−0.11)	0.6 (0.08)	0.019 (−0.03)	0.49 (−0.1)	0.92 (−0.01)

*p* = Mann–Whitney test comparing effects of antibiotics (ABO); r = effect size.

**Table 5:** Effects of delivery mode on fecal butyric acid concentrations.

Stage	Meconium (P1)	7 Days (P2)	1 Month (P3)	6 Months (P4)	12 Months (P5)	24 Months (P6)
Cesarean section	*n* = 13	*n* = 20	*n* = 25	*n* = 38	*n* = 37	*n* = 29
All butyric acid (µmol/g) median (range)	15.89 (2.36–192.5)	15.1 (1.61–210.13)	43.88 (4.63–210.86)	52.17 (9.68–172.87)	106.84 (8.66–341.71)	129.72 (17.17–372.79)
Natural Delivery	*n* = 7	*n* = 11	*n* = 16	*n* = 21	*n* = 19	*n* = 16
All butyric acid (µmol/g) median (range)	12.3 (1.7–99.71)	9.95 (3.08–61.34)	13.49 (2.37–325.46)	34.78 (5.42–229.39)	105.82 (5.14–269.74)	123.01 (47.25–224.64)
*p*, r	0.31 (0.23)	0.16 (0.26)	0.037 (0.32)	0.24 (0.15)	0.78 (−0.04)	0.34 (0.14)

*p* = Mann–Whitney test comparing effects of delivery type; r = effect size.

**Table 6:** Effects of feeding type on linear butyric acid concentration.

Stage	1 Month (P3)	6 Months (P4)	12 Months (P5)	24 Months (P6)
Breastfeeding	*n* = 30	*n* = 26	*n* = 9	*n* = 3
Linear butyric acid (µmol/g) median (range)	21.26 (2.18–313.87)	30.21 (5.34–185.35)	68.34 (2.54–123.9)	114.11 (44.49–183.73)
Formula	*n* = 10	*n* = 32	*n* = 46	*n* = 41
Linear butyric acid (µmol/g) median (range)	35.43 (2.74–101.58)	50.07 (5.6–222.33)	104.07 (4.39–333.58)	111.34 (14.9–357.45)
*p*, r	0.77 (0.04)	0.29 (−0.14)	0.049 (−0.27)	0.80 (−0.04)

*p* = Mann–Whitney test comparing effects of feeding; r = effect size.

**Table 7:** Effect of BMI before pregnancy on all butyrate content.

Stage	Meconium (P1)	7 Days (P2)	1 Month (P3)	6 Months (P4)	12 Months (P5)	24 Months (P6)
BMI before pregnancy 18.5–24.99	*n* = 10	*n* = 12	*n* = 18	*n* = 32	*n* = 28	*n* = 26
All butyric acid (µmol/g) median (range)	12.21 (2.36–192.5)	13.55 (5.76–210.13)	31.49 (4.63–210.86)	59.74 (9.68–229.39)	90.99 (5.28–341.71)	135.92 (47.25–372.79)
BMI before pregnancy < 18.5	*n* = 1	*n* = 5	*n* = 4	*n* = 7	*n* = 6	*n* = 3
All butyric acid (µmol/g) median (range)	154.25 (154.25–154.25)	7.12 (3.08–41.4)	9.91 (2.97–20.44)	18.56 (11.52–55.62)	98.44 (41.68–224.28)	138.49 (86.02–217.54)
*p*, r	1.00 (0.00)	0.08 (0.42)	0.07 (0.39)	0.01 (0.41)	0.46 (−0.13)	0.97 (0.01)

*p* = Mann–Whitney test comparing effects of weight gain; r = effect size.

**Table 8:** Effect of weight gain during pregnancy on all SCFA concentrations.

Stage	Meconium (P1)	7 Days (P2)	1 Month (P3)	6 Months (P4)	12 Months (P5)	24 Months (P6)
Inadequate	*n* = 3	*n* = 7	*n* = 9	*n* = 10	*n* = 9	*n* = 12
All SCFA (µmol/g) median (range)	433.05 (22.36–637.63)	184.78 (19.64–507.52)	214.55 (149.82–621.4)	340.92 (199.34–595.13)	523.65 (67.32–637.63)	360.54 (270.29–520.16)
Adequate	*n* = 4	*n* = 4	*n* = 8	*n* = 12	*n* = 10	*n* = 6
All SCFA (µmol/g) median (range)	97.56 (45.99–497.37)	181.49 (94.15–231.66)	217.44 (67.32–475.85)	333.24 (73.7–551.35)	388.26 (188.92–885.64)	527.02 (401.27–633.96)
Excessive	*n* = 11	*n* = 17	*n* = 21	*n* = 33	*n* = 33	*n* = 25
All SCFA (µmol/g) median (range)	113.09 (16.5–568.9)	183.21 (54.97–610.01)	284.36 (139.93–649.06)	329.09 (29.27–894.84)	434.71 (106.55–687.87)	482.05 (294.96–1163.73)
*p*, r	0.74 (0.04)	0.89 (0.01)	0.42 (0.05)	0.96 (0.001)	0.66 (0.02)	0.02 (0.18)

*p* = Mann–Whitney test comparing effects of weight gain; r = effect size. Weight gain categories: Inadequate (1): <12.5 kg (pre-pregnancy BMI < 18.5 kg/m^2^), <11.5 kg (BMI 18.5–23.9 kg/m^2^), <7 kg (BMI 24.0–27.9 kg/m^2^), and <5 kg (BMI > 28 kg/m^2^); Adequate (1): 12.5–18 kg (BMI < 18.5 kg/m^2^), 11.5–16 kg (BMI 18.5–23.9 kg/m^2^), 7–11.5 kg (BMI 24.0–27.9 kg/m^2^), and 5–9 kg (BMI > 28 kg/m^2^); Excessive (1): >18 kg (BMI < 18.5 kg/m^2^), >16 kg (BMI 18.5–23.9 kg/m^2^), >11.5 kg (BMI 24.0–27.9 kg/m^2^) and >9 kg (BMI > 28 kg/m^2^), according to the 2009 Institute of Medicine/National Research Council GWG recommendations [24].

**Table 9:** Acetate content over time by sex.

Stage	Meconium (P1)	7 Days (P2)	1 Month (P3)	6 Months (P4)	12 Months (P5)	24 Months (P6)
Female	*n* = 11	*n* = 14	*n* = 19	*n* = 24	*n* = 26	*n* = 23
Acetic acid (µmol/g) median (range)	67.33 (12.42–328.8)	80.66 (32.24–430.51)	175.9 (55.12–466.02)	203.24 (74.82–435.63)	227.25 (84.6–445.09)	225 (114.7–686.93)
Male	*n* = 9	*n* = 17	*n* = 22	*n* = 35	*n* = 30	*n* = 22
Acetic acid (µmol/g) median (range)	96.69 (32.46–393.51)	144.5 (4.11–264.67)	186.62 (72.87–337.3)	226.1 (12.55–516.45)	233.6 (55.12–511.2)	187.01 (121.42–304.82)
*p*, r	0.26 (−0.25)	0.19 (−0.24)	0.95 (−0.01)	0.77 (−0.04)	0.85 (−0.02)	0.04 (0.31)

*p* = Mann–Whitney test comparing effects of sex; r = effect size.

**Table 10:** Summary of the multivariable and univariable analyses of SCFAs.

Response/ Factors	ABO	Delivery Mode	Feeding Type	BMI before Pregnancy	Weight Gain	Sex
All SCFA	P1_U_, P4_U_		P5_M_		P6_UM_	P6_M_
Acetic	P1_U_, P4_U_					P6_U_
Propionic	P1_U_, P4_U_			P6_M_	P6_M_	
Linear butyric		P3_U_	P5_UM_	P2_U_, P4_UM_	P6_UM_	P6_M_
Branched butyric		P2_UM_, P5_M_		P5_M_	P2_M_, P6_UM_	
All butyric		P3_U_	P5_M_	P4_UM_	P6_UM_	P6_M_

## Data Availability

Data are available upon request.

## Supplementary Materials

The following supporting information can be downloaded at: https://www.mdpi.com/article/10.3390/nu15020367/s1, Table S1. Acetic acid concentrations (median) in children over time. Table S2. Propionic acid concentrations (median) in children over time. Table S3. Branched butyric acid concentrations (median) in children over time. Table S4. Linear butyric acid concentrations (median) in children over time. Table S5. All SCFA concentrations (median) in children over time. Table S6. Effects of antibiotic therapy during pregnancy on fecal acetic acid concentrations. Table S7. Effects of antibiotic therapy during pregnancy on propionic acid concentrations. Table S8. Effects of antibiotic therapy during pregnancy on SCFA concentrations (µmol/g), median (range). Table S9. Effects of antibiotic therapy of mothers during delivery on SCFA concentrations (µmol/g), median (range). Table S10. The effect of delivery method and antibiotic therapy at delivery on SCFA concentrations (µmol/g), median (range). Table S11. Effects of antibiotic therapy during pregnancy or delivery on SCFA concentrations (µmol/g), median (range). Table S12. Effects of antibiotic therapy during pregnancy, delivery or in childhood on SCFA concentrations (µmol/g), median (range). Table S13. Effects of delivery mode on branched butyrate concentration. Table S14. Effects of delivery mode on linear butyrate concentration. Table S15. Effects of mode of delivery on SCFA concentrations (µmol/g), median (range). Table S16. Effects of type of feeding on SCFA concentrations (µmol/g), median (range). Table S17. Effects of type of feeding of children born naturally on SCFA concentrations (µmol/g), median (range). Table S18. Effects of type of feeding of children born via Cesarean section on SCFA concentrations (µmol/g), median (range). Table S19. Effect of BMI before pregnancy on linear butyric acid concentration. Table S20. Effects of BMI before pregnancy on SCFA concentrations (µmol/g), median (range). Table S21. Effect of weight gain during pregnancy on branched chain butyric acid concentration. Table S22. Effect of weight gain during pregnancy on all butyrate content. Table S23. Effects of weight gain during pregnancy on SCFA concentrations (µmol/g), median (range). Table S24. Effects of children body mass on SCFA concentrations (µmol/g), median (range). Table S25. Effect of birth weight on SCFA concentrations (µmol/g), median (range). Table S26. Effects of sex on SCFA concentrations (µmol/g), median (range). Figure S1. Acetic acid concentrations (median) in children over time of mothers with (red line) or without (blue line) antibiotic therapy during pregnancy (ABO). Figure S2. Propionic acid concentrations (median) in children over time of mothers with (red line) or without (blue line) antibiotic therapy during pregnancy (ABO). Figure S3. Branched butyric acid concentrations (median) in children over time according to delivery type: Cesarean section (blue line) or vaginal birth (red line). Figure S4. Linear butyric acid concentrations (median) in children over time according to delivery type: Cesarean section (blue line) or vaginal birth (red line). Figure S5. Linear butyric acid (median) in children over time according to the effects of BMI before pregnancy: BMI before pregnancy 18.5–24.99 or BMI before pregnancy < 18.5. Figure S6. Branched butyric acid (median) in children over time according to the effects of weight gain during pregnancy: Excessive, Adequate or Inadequate. Figure S7. All butyric acid (median) in children over time according to the effects of weight gain during pregnancy: Excessive, Adequate or Inadequate.
