# Supplementary material for: Analysis of Fecal Short-Chain Fatty Acids (SCFAs) in Healthy Children during the First Two Years of Life: An Observational Prospective Cohort Study
Source: Nutrients. 2023 Jan 11;15(2):367. doi: 10.3390/nu15020367 (PMC9864378; doi:10.3390/nu15020367)
Supplement: Supplementary file 1 [file nutrients-15-00367-s001.zip › nutrients-2076724-supplementary.pdf]

SUPPLEMENTARY MATERIALS

Analysis of faecal short chain fatty acids (SCFA) in healthy children during the first two years of life. An Observational Prospective Cohort Study

Beata Łoniewska[1], Magda Fraszczyk-Tousty[1], Piotr Tousty[2], Karolina Skonieczna-Żydecka[3]*, Dominika Maciejewska-Markiewicz[4], Igor Łoniewski[3,5]

[1] Department of Neonatology and Intensive Neonatal Care, Pomeranian Medical University in Szczecin, Szczecin, Poland; beata.loniewska@pum.edu.pl

[2] Department of Obstetrics and Gynecology, Pomeranian Medical University in Szczecin, Szczecin, Poland; piotr.tousty@pum.edu.pl

[3] Department of Biochemical Science, Pomeranian Medical University in Szczecin, Szczecin, Poland; karzyd@pum.edu.pl

[4] Department of Human Nutrition and Metabolomics, Pomeranian Medical University in Szczecin, Poland; dmaciejewska.pum@gmail.com

[5] Sanprobi sp. z o.o. sp. k. Szczecin, Poland; sanprobi@sanprobi.pl

* Correspondence: karzyd@pum.edu.pl; Tel.:+48 91 441 4808

CONTENT

SUPPLEMENTARY TABLES

Table S 1. Acetic acid concentrations (median) in children over time 3
Table S 2. Propionic acid concentrations (median) in children over time. 4
Table S 3. Branched butyric acid concentrations (median) in children over time. 5
Table S 4. Linear butyric acid concentrations (median) in children over time. 6
Table S 5. All SCFA concentrations (median) in children over time. 7
Table S 6. Effects of antibiotic therapy during pregnancy on faecal acetic acid concentrations. 8
Table S 7. Effects of antibiotic therapy during pregnancy on propionic acid concentrations. 9
Table S 8. Effects of antibiotic therapy during pregnancy on SCFA concentrations (μmol/g), median (range). 10
Table S 9. Effects of antibiotic therapy of mothers during delivery on SCFA concentrations (μmol/g), median (range). 11
Table S 10. The effect of delivery method and antibiotic therapy at delivery on SCFA concentrations (μmol/g), median (range). 13
Table S 11. Effects of antibiotic therapy during pregnancy or delivery on SCFA concentrations (μmol/g), median (range). 15
Table S 12.Effects of antibiotic therapy during pregnancy, delivery or in childchood on SCFA concentrations (μmol/g), median (range). 17
Table S 13. Effects of delivery mode on branched butyrate concentration. 19
Table S 14. Effects of delivery mode on linear butyrate concentration. 20
Table S 15. Effects of mode of delivery on SCFA concentrations (μmol/g), median (range). 21
Table S 16. Effects of type of feeding on SCFA concentrations (μmol/g), median (range). 23
Table S 17. Effects of type of feeding of children born naturally on SCFA concentrations (μmol/g), median (range). 25
Table S 18. Effects of type of feeding of children born via caesarean section on SCFA concentrations (μmol/g), median (range). 26
Table S 19. Effect of BMI before pregnancy on linear butyric acid concentration. 28
Table S 20. Effects of BMI before pregnancy on SCFA concentrations (μmol/g), median (range). 29
Table S 21. Effect of weight gain during pregnancy on branched chain butyric acid concentration. 35
Table S 22.Effect of weight gain during pregnancy on all butyrate content. 36
Table S 23. Effects of weight gain during pregnancy on SCFA concentrations (μmol/g), median (range) 37

Table S 24. Effects of children body mass on SCFA concentrations (μmol/g), median (range). 40
Table S 25. Effect of birth weight on SCFA concentrations (μmol/g), median (range). 42
Table S 26. Effects of sex on SCFA concentrations (μmol/g), median (range). 44

SUPPLEMENTARY FIGURES

Figure S 1. Acetic acid concentrations (median) in children over time of mothers with (red line) or without (blue line) antibiotic therapy during pregnancy (ABO). 46
Figure S 2. Propionic acid concentrations (median) in children over time of mothers with (red line) or without (blue line) antibiotic therapy during pregnancy(ABO). 47
Figure S 3. Branched butyric acid concentrations (median) in children over time with delivery type: caesarean section (blue line) or vaginal birth (red line)...................................... 48
Figure S 4. Linear butyric acid concentrations (median) in children over time with delivery type: caesarean section (blue line) or vaginal birth (red line). 49
Figure S 5. Linear butyric acid (median) in children over time with effects of BMI before pregnancy: BMI before pregnancy 18.5-24.99 (red line) or BMI before pregnancy <18.5 (blue line). 50
Figure S 6. Branched butyric acid (median) in children over time with effects of weight gain during pregnancy Excessive (blue line), Adequate (red line) or inadequate (green line). 51
Figure S 7. All butyric acid (median) in children over time with effects of weight gain during pregnancy Excessive (blue line), Adequate (red line) or inadequate (green line). 52

Table S 1. Acetic acid concentrations (median) in children over time.

	Meconium	7 days	1 month	6 months	12 months	24 months
**Concentrations (µmol/g)**						
	**n=20**	**n=31**	**n=41**	**n=59**	**n=56**	**n=45**
**Median (range)**	88.49 (12.43-393.52)	129.27 (4.12-430.51)	182.22 (55.13-466.02)	213.17 (12.56-516.45)	233.49 (55.13-511.2)	207.5 (114.7-686.93)
**Statistical analyses (Upper-right: differences; Lower-left: correlations)**						
**Meconium**	X	**n=9**	**n=9**	**n=13**	**n=15**	**n=15**
		p=0.03 r=0.73	p=0.08 r=0.57	p=0.007 r=0.75	p=0.002 r=0.78	p=0.006 r=0.7
**7 days**	**n=9**	X	**n=17**	**n=24**	**n=24**	**n=19**
	R=0.71 p=0.03		p=0.02 r=0.56	p=0.004 r=0.58	p=0.001 r=0.73	p=0.01 r=0.58
**1 month**	**n=9**	**n=17**	X	**n=33**	**n=30**	**n=34**
	R=0.28 p=0.46	R=-0.03 p=0.9		p=0.17 r=0.23	p=0.18 r=0.24	p=0.42 r=0.16
**6 months**	**n=13**	**n=24**	**n=33**	X	**n=45**	**n=38**
	R=-0.02 p=0.94	R=0.11 p=0.59	R=0.08 p=0.65		p=0.43 r=0.12	p=0.5 r=0.08
**12 months**	**n=15**	**n=24**	**n=30**	**n=45**	X	**n=30**
	R=0.34 p=0.21	R=0.1 p=0.62	R=-0.06 p=0.71	R=0.26 p=0.07		p=0.7 r=0.07
**24 months**	**n=15**	**n=19**	**n=24**	**n=38**	**n=30**	X
	R=-0.04 p=0.88	R=0.41 p=0.08	R=0.02 p=0.91	R=0.34 p=0.03	R=0.31 p=0.1	

n - number of observations, R - correlation coefficient (Spearman's), p - statistical significance (Wilcoxon signed rank test), r - effect size;

Table S 2. Propionic acid concentrations (median) in children over time.

	Meconium	7 days	1 month	6 months	12 months	24 months
**Concentrations (µmol/g)**						
	**n=20**	**n=31**	**n=41**	**n=59**	**n=56**	**n=45**
**Median (range)**	1.94 (1.57-234.5)	14.72 (4.76-61.74)	32.87 (5.08-106.6)	55.69 (3.16-210.7)	87.49 (2.6-187.6)	86.36 (14.58-285.46)
**Statistical analyses (Upper-right: differences; Lower-left: correlations)**						
		**n=9**	**n=9**	**n=13**	**n=15**	**n=15**
**Meconium**	X	p=0.03 r=0.69	p=0.77 r=0.1	p=0.02 r=0.61	p=0.01 r=0.62	p=0.001 r=0.81
	**n=9**		**n=17**	**n=24**	**n=24**	**n=19**
**7 days**	R=0.45 p=0.22	X	p=0.11 r=0.38	p=0.001 r=0.71	p=0.001 r=0.86	p=0.001 r=0.83
	**n=9**	**n=17**		**n=33**	**n=30**	**n=24**
**1 month**	R=0.6 p=0.08	R=0.33 p=0.19	X	p=0.005 r=0.48	p=0.001 r=0.58	p=0.001 r=0.73
	**n=13**	**n=24**	**n=33**		**n=45**	**n=38**
**6 months**	R=-0.25 p=0.4	R=0.29 p=0.16	R=0.21 p=0.23	X	p=0.04 r=0.3	p=0.02 r=0.36
	**n=15**	**n=24**	**n=30**	**n=45**		**n=29**
**12 months**	R=-0.41 p=0.12	R=0.22 p=0.29	R=-0.23 p=0.22	R=-0.07 p=0.61	X	p=0.11 r=0.29
	**n=15**	**n=19**	**n=24**	**n=38**	**n=30**	
**24 months**	R=-0.4 p=0.13	R=-0.21 p=0.38	R=0.13 p=0.54	R=0.04 p=0.79	R=0.17 p=0.37	X

n - number of observations, R - correlation coefficient (Spearman's), p - statistical significance (Wilcoxon signed rank test), r - effect size;

Table S 3. Branched butyric acid concentrations (median) in children over time.

	Meconium	7 days	1 month	6 months	12 months	24 months
**Concentrations (μmol/g)**						
**Median (range)**	**n=20**	**n=31**	**n=41**	**n=59**	**n==56**	**n=45**
	5.82 (0.08-94.75)	1.95 (0.09-35.71)	3.25 (0.19-72.18)	3.26 (0.08-31.6)	5.53 (0.59-42.33)	11.3 (1.27-140.25)
**Statistical analyses (Upper-right: differences; Lower-left: correlations)**						
**Meconium**	X	**n=9**	**n=9**	**n=13**	**n=15**	**n=15**
		p=0.86 r=0.06	p=0.86 r=0.06	p=0.46 r=0.2	p=0.23 r=0.31	p=0.14 r=0.38
**7 days**	**n=9**	X	**n=17**	**n=24**	**n=24**	**n=19**
	R=0.03 p=0.93		p=0.72 r=0.09	p=0.16 r=0.29	p=0.02 r=0.45	p=0.002 r=0.7
**1 month**	**n=9**	**n=17**	X	**n=33**	**n=30**	**n=24**
	R=0.08 p=0.83	R=0.22 p=0.38		p=0.34 r=0.17	p=0.38 r=0.16	p=0.001 r=0.7
**6 months**	**n=13**	**n=24**	**n=33**	X	**n=45**	**n=38**
	R=0.52 p=0.06	R=0.46 p=0.02	R=0.18 p=0.31		p=0.74 r=0.05	p=0.001 r=0.53
**12 months**	**n=15**	**n=24**	**n=30**	**n=45**	X	**n=30**
	R=0.11 p=0.7	R=0.31 p=0.13	R=0.35 p=0.06	R=0.17 p=0.25		p=0.002 r=0.54
**24 months**	**n=15**	**n=19**	**n=24**	**n=38**	**n=30**	X
	R=0.30 p=0.28	R=0.30 p=0.21	R=0.45 p=0.03	R=0.36 p=0.02	R=0.32 p=0.08	

n - number of observations, R - correlation coefficient (Spearman's), p - statistical significance (Wilcoxon signed rank test), r - effect size;

Table S 4. Linear butyric acid concentrations (median) in children over time.

	Meconium	7 days	1 month	6 months	12 months	24 months
**Concentrations (µmol/g)**						
	**n=20**	**n=31**	**n=41**	**n=59**	**n=56**	**n=45**
**Median (range)**	8.96 (1.2-191.3)	11.06 (1.48-195.52)	22.96 (2.19-313.88)	45.9 (5.35-222.34)	95.2 (2.54-333.58)	111.34 (14.9-357.45)
**Statistical analyses (Upper-right: differences; Lower-left: correlations)**						
**Meconium**	X	**n=9**<br>p=0.051<br>r=0.65	**n=9**<br>p=0.11<br>r=0.53	**n=13**<br>p=0.02<br>r=0.65	**n=15**<br>p=0.003<br>r=0.76	**n=15**<br>p=0.003<br>r=0.75
**7 days**	**n=9**<br>R=0.55<br>p=0.12	X	**n=17**<br>p=0.55<br>r=0.14	**n=24**<br>p=0.003<br>r=0.6	**n=24**<br>p=0.001<br>r=0.87	**n=19**<br>p=0.001<br>r=0.78
**1 month**	**n=9**<br>R=0.93<br>p=0.001	**n=17**<br>R=-0.02<br>p=0.92	X	**n=33**<br>p=0.02<br>r=0.42	**n=30**<br>p=0.006<br>r=0.5	**n=24**<br>p=0.007<br>r=0.55
**6 months**	**n=13**<br>R=-0.21<br>p=0.47	**n=24**<br>R=0.27<br>p=0.2	**n=33**<br>R=0.41<br>p=0.01	X	**n=45**<br>p=0.001<br>r=0.56	**n=38**<br>p=0.001<br>r=0.65
**12 months**	**n=15**<br>R=-0.18<br>p=0.51	**n=24**<br>R=-0.06<br>p=0.76	**n=30**<br>R=-0.21<br>p=0.25	**n=45**<br>R=-0.09<br>p=0.54	X	**n=30**<br>p=0.18<br>r=0.24
**24 months**	**n=15**<br>R=0.13<br>p=0.66	**n=19**<br>R=0.36<br>p=0.12	**n=24**<br>R=-0.22<br>p=0.29	**n=38**<br>R=0.03<br>p=0.86	**n=30**<br>R=-0.07<br>p=0.67	X

n - number of observations, R - correlation coefficient (Spearman's), p - statistical significance (Wilcoxon signed rank test), r - effect size;

Table S 5. All SCFA concentrations (median) in children over time.

	Meconium	7 days	1 month	6 months	12 months	24 months
**Concentrations (μmol/g)**						
	**n=20**	**n=31**	**n=41**	**n=59**	**n=56**	**n=45**
**Median (range)**	129.84 (24.82-775.04)	198.86 (32.72-621.26)	280.86 (69.47-703.16)	357.22 (33.68-910.28)	458.6 (69.47-912.23)	488.14 (280.31-1291.25)
**Statistical analyses (Upper-right: differences; Lower-left: correlations)**						
**Meconium**	X	**n=9**	**n=9**	**n=13**	**n=15**	**n=15**
		p=0.02 r=0.77	p=0.11 r=0.53	p=0.01 r=0.69	p=0.004 r=0.73	p=0.001 r=0.81
**7 days**	**n=9**	X	**n=17**	**n=24**	**n=24**	**n=19**
	R=0.63 p=0.06		p=0.02 r=0.57	p=0.003 r=0.61	p=0.001 r=0.86	p=0.001 r=0.85
**1 month**	**n=9**	**n=17**	X	**n=33**	**n=30**	**n=24**
	R=0.56 p=0.11	R=-0.08 p=0.73		p=0.03 r=0.38	p=0.02 r=0.43	p=0.002 r=0.62
**6 months**	**n=13**	**n=24**	**n=33**	X	**n=45**	**n=38**
	R=0.01 p=0.97	R=0.08 p=0.7	R=0.22 p=0.21		p=0.009 r=0.39	p=0.007 p=0.43
**12 months**	**n=15**	**n=24**	**n=30**	**n=45**	X	**n=30**
	R=0.01 p=0.95	R=0.14 p=0.51	R=-0.31 p=0.09	R=0.1 p=0.5		p=0.18 r=0.25
**24 months**	**n=15**	**n=19**	**n=24**	**n=38**	**n=30**	X
	R=-0.06 p=0.81	R=0.6 p=0.006	R=0.04 p=0.83	R=0.06 p=0.71	R=0.14 p=0.45	

n - number of observations, R - correlation coefficient (Spearman's), p - statistical significance (Wilcoxon signed rank test), r - effect size;

Table S 6. Effects of antibiotic therapy during pregnancy on faecal acetic acid concentrations.

Stage	Meconium(P1)	7 days(P2)	1 month(P3)	6 months(P4)	12 months(P5)	24 months(P6)
**ABO YES**	n=6	n=8	n=10	n=13	n=17	n=9
**Acetic acid(µmol/g) median(range)**	212.93 (86.0-328.8)	108.4 (69.61-430.51)	157.57 (110.4-466.02)	175.52 (62.61-287.5)	195.77 (84.6-428.3)	225 (140.8-388.07)
**ABO NO**	n=14	n=23	n=31	n=46	n=39	n=36
**Acetic acid(µmol/g) median(range)**	66.22 (12.42-393.51)	144.50 (4.11-372.42)	191.04 (55.12-383.74)	234.64 (12.55-516.45)	233.67 (55.12-511.2)	203.40 (114.7-686.93)
**p, r**	0.02 (0.51)	0.46(-0.13)	0.94(-0.01)	0.04(-0.26)	0.34(-0.13)	0.34(0.14)

p - Mann–Whitney test comparing effects of antibiotics (ABO), r - effect size.

Table S 7. Effects of antibiotic therapy during pregnancy on propionic acid concentrations.

Stage	Meconium (P1)	7 days (P2)	1 month (P3)	6 months (P4)	12 months (P5)	24 months (P6)
**ABO YES**	n=6	n=8	n=10	n=13	n=17	n=9
**Propionic acid(µmol/g) median(range)**	60.12 (14.97-234.5)	15.04 (5.1-52.7)	26.13 (10.51-84.31)	36.98 (3.16-75.84)	66.6 (6.09-187.6)	69.51 (47.95-150.62)
**ABO NO**	n=14	n=23	n=31	n=46	n=39	n=36
**Propionic acid(µmol/g) median(range)**	10.01 (1.57-117.6)	14.71 (4.76-61.74)	33.61 (5.07-106.6)	62.65 (5.73-210.69)	89.87 (2.6-180.1)	94.93 (14.58-285.45)
**p, r**	0.006(0.58)	0.98(0.001)	1.00(0.00)	0.007(-0.34)	0.846(-0.03)	0.076(-0.26)

p - Mann–Whitney test comparing effects of antibiotics (ABO), r - effect size

Table S 8. Effects of antibiotic therapy during pregnancy on SCFA concentrations (μmol/g), median (range).

	Antibiotic therapy during pregnancy		
	Yes	No	p/r
**Branched butyric acid at meconium**	n=6	n=14	0.153/0.32
	7.02(3.69-20.64)	1.77(0.08-94.75)	
**Branched butyric acid at 7 days**	n=8	n=23	0.674/0.08
	2.45(0.56-8.01)	1.95(0.09-35.71)	
**Branched butyric acid at 1 month**	n=10	n=31	0.345/0.15
	3.84(0.89-72.18)	2.75(0.19-52.15)	
**Branched butyric acid at 6 months**	n=13	n=46	0.227/-0.16
	3.04(0.08-20.66)	3.38(1.16-31.6)	
**Branched butyric acid at 12 months**	n=17	n=39	0.447/0.10
	6.26(0.82-42.33)	5.02(0.59-35.19)	
**Branched butyric acid 24 months**	n=9	n=36	0.459/-0.11
	6.84(1.41-31.6)	12.32(1.27-140.20)	
**Linear butyric acid at meconium**	n=6	n=14	0.274/0.25
	18.51(5.64-156.82)	8.88(1.2-191.3.00)	
**Linear butyric acid at 7 days**	n=8	n=23	0.740/0.06
	10.53(6.48-59.59)	11.43(1.48-195.52)	
**Linear butyric acid at 1 month**	n=10	n=31	0.376/0.14
	39.91(2.74-156.48)	19.56(2.19-313.88)	
**Linear butyric acid at 6 months**	n=13	n=46	0.380/-0.12
	17.05(5.35-140.7)	46.03(5.6-222.34)	
**Linear butyric acid at 12 months**	n=17	n=39	0.958/0.01
	93.63(6.29-264.4)	96.77(2.54-333.58)	
**Linear butyric acid at 24 months**	n=9	n=36	0.856/0.03
	115.06(47.7-271.49)	107.5(14.9-357.46)	
**All butyric acid at meconium**	n=6	n=14	0.274/0.25
	24.3(11.13-177.47)	13.37(1.7-192.5)	
**All butyric acid at 7 days**	n=8	n=23	0.740/0.06
	12.61(9.48-61.34)	13.65(1.62-210.13)	
**All butyric acid at 1 month**	n=10	n=31	0.223/0.19
	52.03(7.79-170.4.)	26.98(2.37-325.47)	
**All butyric acid at 6 months**	n=13	n=46	0.314/-0.13
	19.96(5.43-142.68)	48.48(9.68-229.39)	
**All butyric acid at 12 months**	n=17	n=39	0.888/0.02
	124.78(15.86-269.74)	105.82(5.15-341.72)	
**All butyric acid at 24 months**	n=9	n=36	0.967/-0.01
	146.67(54.35-275.89)	128.38(17.17-372.79)	

p - statistical significance, r - effect size

Table S 9. Effects of antibiotic therapy of mothers during delivery on SCFA concentrations (μmol/g), median (range).

	Antibiotic therapy of mothers during delivery		
	Yes	No	p/r
**Acetic acid at meconium**	n=14<br>91.35(17.60-393.52)	n=5<br>65.11(12.43-259.36)	0.487/0.16
**Acetic acid at 7 days**	n=23<br>129.27(61.6-372.42)	n=6<br>143.64(32.24-261.48)	0.893/0.02
**Acetic acid at 1 month**	n=30<br>191.26(71.9-466)	n=8<br>161.52(55.13-250.85)	0.111/0.26
**Acetic acid at 6 months**	n=46<br>210.84(12.56-516.45)	n=9<br>226.1(74.82-360.8)	0.724/-0.05
**Acetic acid at 12 months**	n=42<br>228.78(84.61-445.1)	n=10<br>292.28(147.41-511.2)	0.112/-0.22
**Acetic acid at 24 months**	n=35<br>202.99(114.7-686.93)	n=7<br>207.5(159.2-243.81)	0.813/0.04
**Propionic acid at meconium**	n=14<br>15.87(1.57-234.5)	n=5<br>7.07(2.01-73.97)	0.247/0.27
**Propionic acid at 7 days**	n=23<br>14.72(4.76-61.74)	n=6<br>16.22(5.84-29.23)	0.893/0.02
**Propionic acid at 1 month**	n=30<br>31.78(6.27-106.6)	n=8<br>30.72(5.08-84.59)	0.654/0.07
**Propionic acid at 6 months**	n=46<br>55.2(5.74-180.7)	n=9<br>61.93(19.9-194.22)	0.724/-0.05
**Propionic acid at 12 months**	n=42<br>67.86(2.6-180.1)	n=10<br>102.36(45.52-187.6)	0.050/-0.27
**Propionic acid at 24 months**	n=35<br>86.36(14.58-285.46)	n=7<br>93.84(58.79-151.5)	0.787/-0.04
**Branched butyric acid at meconium**	n=14<br>5.82(0.1-94.75)	n=5<br>6.75(0.08-15.71)	0.817/0.05
**Branched butyric acid at 7 days**	n=23<br>1.97(0.14-35.71)	n=6<br>1.07(0.09-5.06)	0.187/0.24
**Branched butyric acid at 1 month**	n=30<br>2.81(0.26-52.15)	n=8<br>2.02(0.19-72.18)	0.604/0.08
**Branched butyric acid at 6 months**	n=46<br>3.03(0.08-31.6)	n=9<br>3.5(1.51-11.89)	0.547/-0.08
**Branched butyric acid at 12 months**	n=42<br>5.19(0.59-35.19)	n=10<br>5.63(1.98-22.54)	0.410/-0.11
**Branched butyric acid at 24 months**	n=35<br>10.21(1.27-140.25)	n=7<br>13.95(1.92-48.3)	0.478/-0.11
**Linear butyric acid at meconium**	n=14<br>8.88(1.26-191.3)	n=5<br>10.07(1.2-84)	0.746/0.07

**Linear butyric acid at 7 days**	n=23	n=6	0.206/0.23
	11.43(1.48-195.52)	7.23(3-11.83)	
**Linear butyric acid at 1 month**	n=30	n=8	0.119/0.25
	27.09(3.61-209)	7.87(2.19-313.88)	
**Linear butyric acid at 6 months**	n=46	n=9	0.991/0.00
	46.03(5.35-222.34)	58(8.39-185.36)	
**Linear butyric acid at 12 months**	n=42	n=10	0.080/-0.24
	90.6(4.4-333.58)	128.91(29.19-264.4)	
**Linear butyric acid at 24 months**	n=35	n=7	0.787/0.04
	111.34(14.9-357.45)	137.5(44.34-183.74)	
**All SCFA at meconium**	n=14	n=5	0.379/0.20
	132.12(27.13-775.04)	95.92(24.82-444.62)	
**All SCFA at 7 days**	n=23	n=6	0.686/0.07
	198.86(81.37-621.26)	175.74(64.19-301.62)	
**All SCFA at 1 month**	n=30	n=8	0.168/0.22
	302.65(151.47-703.16)	255.3(69.47-655.32)	
**All SCFA at 6 months**	n=46	n=9	0.609/-0.07
	358.47(33.69-910.28)	379.71(196.39-613.4)	
**All SCFA at 12 months**	n=42	n=10	0.062/-0.26
	436.19(115.33-695.24)	572.39(319.95-912.23)	
**All SCFA at 24 months**	n=35	n=7	0.866/0.03
	488.14(280.31-1291.25)	474.07(322.78-616.3)	

p - statistical significance, r - effect size

Table S 10. The effect of delivery method and antibiotic therapy at delivery on SCFA concentrations (μmol/g), median (range).

	SCFA Concentrations in vaginal birth: with (ATB) or without antibiotics (N) or caesarean section (CS)			
	N	ATB	CS	p /r
**Acetic acid at meconium**	n=5	n=1	n=13	0.210/0.17
	65.11(12.43-259.36)	17.6(17.6-17.6)	96.7(19-393.52)	
**Acetic acid at 7 days**	n=6	n=3	n=20	0.960/0.00
	143.64(32.24-261.48)	137.9(80.37-240.86)	126.63(61.6-372.42)	
**Acetic acid at 1 month**	n=8	n=5	n=25	0.260/0.07
	161.52(55.13-250.85)	182.22(113.5-292.8)	200.3(71.9-466)	
**Acetic acid at 6 months**	n=9	n=8	n=38	0.820/0.01
	226.1(74.82-360.8)	184.75(41.78-516.45)	215.79(12.56-435.64)	
**Acetic acid at 12 months**	n=10	n=5	n=37	0.341/-0.02
	292.28(147.41-511.2)	249(150.6-290.8)	223.3(84.6-445)	
**Acetic acid at 24 months**	n=7	n=6	n=29	0.260/0.05
	207.5(159.2-243.81)	216.9(169.6-304.82)	202.99(114.7-686.93)	
**Propionic acid at meconium**	n=5	n=1	n=13	0.560/0.03
	7.07(2-73.97)	1.97(1.97-1.97)	16.78(1.57-234.5)	
**Propionic acid at 7 days**	n=6	n=3	n=20	0.130/0.23
	16.22(5.84-29.23)	9.39(8.1-14.42)	17.83(4.76-61.74)	
**Propionic acid at 1 month**	n=8	n=5	n=25	0.660/0.03
	30.72(5.08-84.6)	22.87(9.6-106.6)	32.87(6.27-104.39)	
**Propionic acid at 6 months**	n=9	n=8	n=38	0.900/0.01
	619.26(199.02-194.22)	43.58(6.77-149)	57.14(5.74-180.7)	
**Propionic acid at 12 months**	n=10	n=5	n=37	0.870/0.01
	102.36(45.52-187.6)	114.1(2.6-164.6)	66.6(6.09-180.1)	
**Propionic acid at 24 months**	n=7	n=6	n=29	0.080/0.10
	93.84(58.79-151.5)	97.19(18.47-179.9)	86.1(14.58-285.46)	
**Branched butyric acid at meconium**	n=5	n=1	n=13	0.800/0.01
	6.75(0.08-15.7)	0.1(0.1-0.1)	6.14(0.24-94.75)	
**Branched butyric acid at 7 days**	n=6	n=3	n=20	0.310/0.13
	1.07(0.09-5.06)	0.56(0.4-1.95)	2.68(0.14-35.71)	
**Branched butyric acid at 1 month**	n=8	n=5	n=25	0.110/0.16
	2.02(0.19-72.19)	4.64(0.69-5.21)	2.37(0.26-52.15)	

**Branched butyric acid at 6 months**	n=9<br>3.5(1.51-11.89)	n=8<br>3.62(0.08-7.54)	n=38<br>2.91(0.33-31.6)	0.840/0.01
**Branched butyric acid at 12 months**	n=10<br>5.63(1.98-22.54)	n=5<br>3.82(0.75-15.7)	n=37<br>5.27(0.59-35.19)	0.800/0.01
**Branched butyric acid at 24 months**	n=7<br>13.95(1.92-48.3)	n=6<br>3.39(1.88-31.6)	n=29<br>11.3(1.27-140.25)	0.680/0.02
**Linear butyric acid at meconium**	n=5<br>10.07(1.2-84)	n=1<br>2.69(2.69-2.69)	n=13<br>9.3(1.26-191.3)	0.530/0.03
**Linear butyric acid at 7 days**	n=6<br>7.23(3.00-11.83)	n=3<br>11.06(3.82-60.28)	n=20<br>11.43(1.48-195.52)	0.380/0.11
**Linear butyric acid at 1 month**	n=8<br>7.87(2.19-313.88)	n=5<br>8.48(5.3-23.75)	n=25<br>37.4(3.61-209)	0.420/0.06
**Linear butyric acid at 6 months**	n=9<br>58(8.39-185.36)	n=8<br>19.6(5.35-222.34)	n=38<br>49.82(5.96-157.4)	0.070/0.14
**Linear butyric acid at 12 months**	n=10<br>128.91(29.19-264.00)	n=5<br>68.34(4.4-157.9)	n=37<br>96.77(4.44-333.58)	0.470/0.03
**Linear butyric acid at 24 months**	n=7<br>137.50(44.34-183.74)	n=6<br>101.61(44.49-160.5)	n=29<br>111.34(14.9-357.45)	0.130/0.08
**All SCFA at meconium**	n=5<br>95.92(24.82-444.62)	n=1<br>271.32(271.32-271.32)	n=13<br>142.53(34.44-775)	0.679/0.02
**All SCFA at 7 days**	n=6<br>175.74(64.19-301.62)	n=3<br>1846.28(1043.14-3239.61)	n=20<br>199.12(81.37-621.26)	0.179/0.19
**All SCFA at 1 month**	n=8<br>255.3(69.47-655.32)	n=5<br>2101.64(1707.55-4232.98)	n=25<br>304.14 (151.47-703.16)	0.901/0.01
**All SCFA at 6 months**	n=9<br>379.71 (196.39-613.4)	n=8<br>3330.48 (857.96-9102.82)	n=38<br>358.47 (33.68-610.94)	0.284/0.07
**All SCFA at 12 months**	n=10<br>572.39(319.95-912.23)	n=5<br>5167.36(1698.97-6531.43)	n=37<br>435.63(115.33-695.24)	0.816/0.01
**All SCFA at 24 months**	n=7<br>474.07(322.78-616.3)	n=6<br>505.91(358.18-605.7)	n=29<br>478.2(280.31-1291.25)	0.168/0.07

p - statistical significance, r - effect size

Table S 11. Effects of antibiotic therapy during pregnancy or delivery on SCFA concentrations (μmol/g), median (range).

	**Antibiotic therapy of mothers during pregnancy or delivery**		
	Yes	No	p/r
**Acetic acid at meconium**	n=15	n=4	0.152/0.33
	96.7(17.6-393.52)	52(12.43-90.98)	
**Acetic acid at 7 days**	n=23	n=5	0.641/-0.09
	124(61.6-372.42)	163.85(32.24-261.48)	
**Acetic acid at 1 month**	n=32	n=6	0.445/0.13
	179.06(71.9-466)	202.54(55.13-250.85)	
**Acetic acid at 6 months**	n=46	n=8	0.676/-0.06
	210.84(12.56-435.64)	235.91(74.82-360.8)	
**Acetic acid at 12 months**	n=44	n=8	0.195/-0.18
	228.78(84.61-445.1)	292.28(147.41-511.2)	
**Acetic acid at 24 months**	n=36	n=5	0.954/-0.01
	200.9(114.7-686.93)	207.5(161.5-243.81)	
**Propionic acid at meconium**	n=15	n=4	0.062/0.42
	16.78(1.57-234.5)	4.72(2-12.18)	
**Propionic acid at 7 days**	n=23	n=5	0.954/0.01
	14.72(4.76-61.74)	18.72(5.84-29.23)	
**Propionic acid at 1 month**	n=32	n=6	0.800/0.04
	29.25(6.27-106.6)	40(5.08-84.59)	
**Propionic acid at 6 months**	n=46	n=8	0.658/-0.06
	55.2(5.74-180.7)	62.65(19.9-194.22)	
**Propionic acid at 12 months**	n=44	n=8	0.233/-0.17
	68.68(2.6-187.6)	97.02(45.52-153.4)	
**Propionic acid at 24 months**	n=36	n=5	0.217/-0.20
	85.26(14.58-285.46)	108.24(81.31-151.5)	
**Branched butyric acid at meconium**	n=15	n=4	0.357/0.22
	6.14(0.1-94.75)	3.63(0.08-9.83)	
**Branched butyric acid at 7 days**	n=23	n=5	0.173/0.26
	1.97(0.14-35.71)	0.59(0.09-5.06)	
**Branched butyric acid at 1 month**	n=32	n=6	0.422/0.13
	2.81(0.26-72.18)	2.02(0.19-11.59)	
**Branched butyric acid at 6 months**	n=46	n=8	0.296/-0.14
	2.97(0.08-31.6)	5.38(1.66-11.89)	
**Branched butyric acid at 12 months**	n=44	n=8	0.756/-0.04
	5.31(0.59-35.19)	5.46(1.98-22.54)	
**Branched butyric acid 24 months**	n=36	n=5	0.265/-0.18
	9.71(1.27-140.25)	21.77(1.92-48.3)	
**Linear butyric acid at meconium**	n=15	n=4	0.357/0.22
	9.3(1.26-191.3)	7.55(1.2-13.83)	
**Linear butyric acid at 7 days**	n=23	n=5	0.348/0.18
	11.06(1.48-195.52)	6.53(3-11.83)	
**Linear butyric acid at 1**	n=32	n=6	0.062/0.30

**month**	27.09(3.61-209)	4.3(2.19-313.88)	
**Linear butyric acid at 6 months**	n=46	n=8	0.952/0.01
	46.03(5.35-157.39)	40.78(8.39-185.36)	
**Linear butyric acid at 12 months**	n=44	n=8	0.223/-0.17
	94.34(4.4-333.58)	122.91(29.19-242.73)	
**Linear butyric acid at 24 months**	n=36	n=5	0.832/0.03
	113.2(14.9-357.45)	137.5(44.34-183.74)	
**All butyric acid at meconium**	n=15	n=4	0.307/0.24
	15.9(2.36-192.5)	10.96(1.7-23.66)	
**All butyric acid at 7 days**	n=23	n=5	0.137/0.28
	13.6(1.62-210.13)	7.12(3.08-16.89)	
**All butyric acid at 1 month**	n=32	n=6	0.062/0.30
	33.57(4.64-210.87)	6.22(2.37-325.47)	
**All butyric acid at 6 months**	n=46	n=8	0.971/0.00
	48.48(5.43-172.87)	48.14(11.53-193.55)	
**All butyric acid at 12 months**	n=44	n=8	0.187/-0.18
	106(5.15-341.72)	129.47(51.73-245.48)	
**All butyric acid at 24 months**	n=36	n=5	0.862/0.03
	134.11(17.17-372.79)	139.42(54.07-224.65)	
**All SCFA at meconium**	n=15	n=4	0.152/0.33
	130.18(22.36-740.77)	67.5(16.51-126.81)	
**All SCFA at 7 days**	n=23	n=5	0.954/-0.01
	163.94(78.3-610.02)	194.35(54.98-293.15)	
**All SCFA at 1 month**	n=32	n=6	0.377/0.15
	278(140-650)	251.11(67.32-632.65)	
**All SCFA at 6 months**	n=46	n=8	0.590/-0.07
	340.92(29.28-580.36)	330.84(183.07-601.42)	
**All SCFA at 12 months**	n=44	n=8	0.187/-0.18
	431.71(106.56-885.64)	562.18(294-867.37)	
**All SCFA at 24 months**	n=36	n=5	0.685/-0.07
	438.96(209.65-1163.73)	498.42(312.87-566.28)	

p - statistical significance, r - effect size

Table S 12. Effects of antibiotic therapy during pregnancy, delivery or in childchood on SCFA concentrations (µmol/g), median (range).

	Antibiotic therapy of mothers during pregnancy or delivery or in childchood		
	Yes	No	p/r
**Acetic acid at meconium**	n=16	n=3	0.085/0.40
	93.84(17.6-393.52)	38.89(12.43-65.11)	
**Acetic acid at 7 days**	n=24	n=4	0.874/-0.03
	126.63(61.6-372.42)	146.2(32.24-261.48)	
**Acetic acid at 1 month**	n=32	n=6	0.445/0.13
	179.06(71.9-466)	202.54(55.13-250.85)	
**Acetic acid at 6 months**	n=47	n=7	0.782/-0.04
	213.17(12.56-435.64)	212.66(74.82-360.8)	
**Acetic acid at 12 months**	n=45	n=7	0.278/-0.15
	233.32(84.61-445.1)	297.36(147.41-511.2)	
**Acetic acid at 24 months**	n=37	n=4	0.719/0.06
	202.99(114.7-686.93)	186.23(161.5-233.4)	
**Propionic acid at meconium**	n=16	n=3	0.064/0.42
	15.87(1.57-234.5)	2.38(2-7.07)	
**Propionic acid at 7 days**	n=24	n=4	0.590/0.11
	15.49(4.76-61.74)	13.28(5.84-24.55)	
**Propionic acid at 1 month**	n=32	n=6	0.800/0.04
	29.25(6.27-106.6)	40(5.08-84.59)	
**Propionic acid at 6 months**	n=47	n=7	0.860/0.02
	55.69(5.74-194.22)	61.93(19.9-85.64)	
**Propionic acid at 12 months**	n=45	n=7	0.290/-0.15
	69.31(2.6-187.6)	95.27(45.52-153.4)	
**Propionic acid at 24 months**	n=37	n=4	0.111/-0.25
	84.41(14.58-285.46)	124.78(93.84-151.5)	
**Branched butyric acid at meconium**	n=16	n=3	0.138/0.35
	6.88(0.1-94.75)	0.5(0.08-6.75)	
**Branched butyric acid at 7 days**	n=24	n=4	0.082/0.33
	2.09(0.14-35.71)	0.35(0.09-5.06)	
**Branched butyric acid at 1 month**	n=32	n=6	0.422/0.13
	2.81(0.26-72.18)	2.02(0.19-11.59)	
**Branched butyric acid at 6 months**	n=47	n=7	0.495/-0.09
	3.02(0.08-31.6)	3.5(1.66-11.89)	
**Branched butyric acid at 12 months**	n=45	n=7	0.979/0.00
	5.34(0.59-35.19)	5.02(1.98-22.54)	
**Branched butyric acid 24 months**	n=37	n=4	0.457/-0.12
	10.21(1.27-140.25)	18.75(1.92-48.3)	
**Linear butyric acid at meconium**	n=16	n=3	0.211/0.29
	11.57(1.26-191.3)	5.03(1.2-10.07)	
**Linear butyric acid at 7 days**	n=24	n=4	0.209/0.24
	11.25(1.48-195.52)	6.53(3-11.83)	

**Linear butyric acid at 1 month**	n=32	n=6	0.062/0.30
	27.09(3.61-209)	4.3(2.19-313.88)	
**Linear butyric acid at 6 months**	n=47	n=7	0.880/-0.02
	45.9(5.35-157.4)	58(8.39-185.36)	
**Linear butyric acid at 12 months**	n=45	n=7	0.303/-0.15
	96.77(4.4-333.58)	104.3(29.19-242.73)	
**Linear butyric acid at 24 months**	n=37	n=4	0.719/-0.06
	111.34(14.9-357.45)	156.93(44.34-183.74)	
**All butyric acid at meconium**	n=16	n=3	0.138/0.35
	19.76(2.36-192.5)	5.1(1.7-16.82)	
**All butyric acid at 7 days**	n=24	n=4	0.070/0.34
	13.62(1.62-210.13)	6.88(3.08-16.89)	
**All butyric acid at 1 month**	n=32	n=6	0.062/0.30
	33.57(4.64-210.87)	6.22(2.37-325.47)	
**All butyric acid at 6 months**	n=47	n=7	0.900/-0.02
	47.71(5.43-172.87)	61.5(11.53-193.55)	
**All butyric acid at 12 months**	n=45	.n=7	0.267/-0.16
	106.84(5.15-341.72)	109.32(51.73-245.48)	
**All butyric acid at 24 months**	n=37	n=4	0.783/-0.04
	129.72(17.17-372.79)	175.47(54.07-224.65)	
**All SCFA at meconium**	n=16	n=3	0.085/0.40
	128.5(22.36-740.77)	46(16.51-88.99)	
**All SCFA at 7 days**	n=24	n=4	0.874/0.03
	173.58(78.31-610)	164.34(54.98-293.15)	
**All SCFA at 1 month**	n=32	n=6	0.377/0.15
	278(139.94-649)	251.11(67.32-632.65)	
**All SCFA at 6 months**	n=47	n=7	0.900/-0.02
	341.3(29.28-580.36)	311.33(183.07-601.42)	
**All SCFA at 12 months**	n=45	n=7	0.244/-0.16
	436.6(106.56-885.64)	588.77(294-867.37)	
**All SCFA at 24 months**	n=37	n=4	0.538/-0.10
	433.41(209.65-1163.73)	506.37(312.87-566.28)	

p - statistical significance, r - effect size

Table S 13. Effects of delivery mode on branched butyrate concentration.

Stage	Meconium(P1)	7 days(P2)	1 month(P3)	6 months(P4)	12 months(P5)	24 months(P6)
**Caesarean section**	n=13	n=20	n=25	n=38	n=37	n=29
**Branched butyric acid(μmol/g) median(range)**	6.14 (0.24-94.75)	2.68 (0.14-35.71)	2.37 (0.26-52.15)	2.91 (0.32-31.6)	5.26 (0.59-35.19)	11.3 (1.26-140.25)
**Natural Delivery**	n=7	n=11	n=16	n=21	n=19	n=16
**Branched butyric acid(μmol/g) median(range)**	3.68 (0.07-15.7)	1.55 (0.08-5.05)	4.05 (0.18-72.18)	4.19 (0.07-14.57)	5.91 (0.75-42.33)	11.13 (1.87-48.29)
**p, r**	0.35(0.21)	0.04(0.35)	0.99(0.001)	0.7(-0.05)	0.38(-0.12)	0.92(-0.02)

p - Mann–Whitney test comparing effects of delivery type, r - effect size

Table S 14. Effects of delivery mode on linear butyrate concentration.

Stage	Meconium(P1)	7 days(P2)	1 month(P3)	6 months(P4)	12 months(P5)	24 months(P6)
**Caesarean section**	n=13	n=20	n=25	n=38	n=37	n=29
**Linear butyric acid(μmol/g) median(range)**	9.3 (1.26-191.3)	11.43 (1.47-195.52)	37.4 (3.6-209.0)	49.81 (5.96-157.39)	96.77 (4.44-333.58)	111.34 (14.9-357.45)
**Natural Delivery**	n=7	n=11	n=16	n=21	n=19	n=16
**Linear butyric acid(μmol/g) median(range)**	8.62 (1.19-84.01)	7.98 (2.99-60.27)	8.37 (2.18-313.87)	22.27 (5.34-222.33)	93.62 (2.54-264.4)	108.81 (29.49-202.3)
**p, r**	0.393(0.19)	0.4(0.15)	0.02(0.36)	0.2(0.17)	0.94(-0.01)	0.43(0.12)

p - Mann–Whitney test comparing effects of delivery type, r - effect size

Table S 15. Effects of mode of delivery on SCFA concentrations (μmol/g), median (range).

	Mode of delivery		
	**Caesarean section**	**Natural**	**p/r**
**Acetic acid at meconium**	n=13	n=7	0.393/0.19
	96.7(19.01-393.52)	65.11(12.43-320.53)	
**Acetic acid at 7 days**	n=20	n=11	-
	126.63(61.6-372.42)	137.9(4.12-430.51)	
**Acetic acid at 1 month**	n=25	n=16	0.162/0.22
	200.3(71.9-466.02)	169.83(55.13-292.8)	
**Acetic acid at 6 months**	n=38	n=21	0.969/0.01
	215.79(12.56-435.64)	212.66(41.78-516.45)	
**Acetic acid at 12 months**	n=37	n=19	0.344/-0.13
	223.3(84.61-445.09)	244.69(55.13-511.2)	
**Acetic acid at 24 months**	n=29	n=16	0.234/-0.18
	202.99(114.7-686.93)	229.19(159.2-388.07)	
**Propionic acid at meconium**	n=13	n=7	0.241/0.27
	16.78(1.57-234.5)	7.07(1.97-164.54)	
**Propionic acid at 7 days**	n=20	n=11	0.381/0.16
	17.83(4.76-61.74)	13.72(5.57-29.23)	
**Propionic acid at 1 month**	n=25	n=16	0.968/0.01
	32.87(6.27-104.39)	30.72(5.08-106.6)	
**Propionic acid at 6 months**	n=38	n=21	0.796/0.03
	57.14(5.74-180.7)	51.06(3.16-210.7)	
**Propionic acid at 12 months**	n=37	n=19	0.066/-0.25
	66.6(6.09-180.1)	101.57(2.6-187.6)	
**Propionic acid at 24 months**	n=29	n=16	0.681/-0.06
	86.11(14.58-285.46)	94.94(18.47-179.9)	
**Branched butyric acid at meconium**	n=13	n=7	0.351/0.21
	6.14(0.24-94.75)	3.69(0.08-15.71)	
**Branched butyric acid at 7 days**	n=20	n=11	0.049/0.35
	2.68(1.40-357.10)	1.55(0.09-5.06)	
**Branched butyric acid at 1 month**	n=25	n=16	0.989/0.00
	2.37(0.26-52.15)	4.06(0.19-72.18)	
**Branched butyric acid at 6 months**	n=38	n=21	0.700/-0.05
	2.91(0.33-31.6)	4.19(0.08-14.57)	
**Branched butyric acid at 12 months**	n=37	n=19	0.381/-0.12
	5.27(0.59-35.19)	5.91(0.75-42.33)	
**Branched butyric acid 24 months**	n=29	n=16	0.916/-0.02
	11.3(1.27-140.25)	11.13(1.88-48.3)	
**Linear butyric acid at meconium**	n=13	n=7	0.393/0.19
	9.31(1.26-191.3)	8.62(1.2-84)	
**Linear butyric acid at 7 days**	n=20	n=11	0.403/0.15
	11.43(1.48-195.52)	7.99(3-60.28)	

**Linear butyric acid at 1 month**	n=25<br>37.4(3.61-209)	n=16<br>8.38(2.19-313.88)	0.020/0.36
**Linear butyric acid at 6 months**	n=38<br>49.82(5.96-157.39)	n=21<br>22.27(5.35-222.34)	0.199/0.17
**Linear butyric acid at 12 months**	n=37<br>96.77(4.44-333.58)	n=19<br>93.63(2.54-264.4)	0.945/-0.01
**Linear butyric acid at 24 months**	n=29<br>111.34(14.9-357.45)	n=16<br>108.81(29.49-202.3)	0.431/0.12
**All butyric acid at meconium**	n=13<br>15.9(2.36-192.5)	n=7<br>12.31(1.7-99.72)	0.311/0.23
**All butyric acid at 7 days**	n=20<br>15.11(1.62-210.13)	n=11<br>9.96(3.08-61.34)	0.157/0.26
**All butyric acid at 1 month**	n=25<br>43.88(4.64-210.87)	n=16<br>13.49(2.37-325.47)	0.037/0.32
**All butyric acid at 6 months**	n=38<br>52.17(9.68-172.87)	n=21<br>34.78(5.43-229.39)	0.240/0.15
**All butyric acid at 12 months**	n=37<br>106.84(8.67-341.72)	n=19<br>105.82(5.15-269.74)	0.784/-0.04
**All butyric acid at 24 months**	n=29<br>129.72(17.17-372.79)	n=16<br>124(47.26-224.65)	0.341/0.14
**All SCFA at meconium**	n=13<br>130.18(29.29-740.77)	n=7<br>88.99(16.51-497.38)	0.311/0.23
**All SCFA at 7 days**	n=20<br>184(78.31-610.02)	n=11<br>163.94(19.64-507.52)	0.555/0.11
**All SCFA at 1 month**	n=25<br>280.32(146.2-649.07)	n=16<br>236.75(67.32-632.65)	0.162/0.22
**All SCFA at 6 months**	n=38<br>341.07(29.28-580.36)	n=21<br>311.33(73.71-894.85)	0.820/0.03
**All SCFA at 12 months**	n=37<br>420.5(106.56-687.87)	n=19<br>447(67.32-885.64)	0.460/-0.10
**All SCFA at 24 months**	n=29<br>447.63(209.65-1163.73)	n=16<br>468.57(294.97-653.57)	0.769/-0.04

p - statistical significance, r - effect size

Table S 16. Effects of type of feeding on SCFA concentrations (μmol/g), median (range).

	Type of feeding		
	**Breast feeding**	**Formula**	**p/r**
**Acetic acid at 1 month**	n=30	n=10	0.229/-0.19
	179.06(55.13-383.75)	234.74(71.9-466)	
**Acetic acid at 6 months**	n=26	n=32	0.772/0.04
	232.15(12.56-435.64)	212.92(41.78-516.45)	
**Acetic acid at 12 months**	n=9	n=46	0.183/-0.18
	1.33(0.2-2.76)	236.76(84.61-511.2)	
**Acetic acid at 24 months**	n=2	n=41	0.840/0.03
	224.86(161.5-288.22)	207.5(114.7-686.93)	
**Propionic acid at 1 month**	n=30	n=10	0.059/-0.30
	27.65(5.08-104.39)	56.97(7.59-106.6)	
**Propionic acid at 6 months**	n=26	n=32	0.749/-0.04
	54.56(6.77-210.7)	56.37(3.16-194.22)	
**Propionic acid at 12 months**	n=9	n=46	0.577/-0.08
	67.68(6.9-152.6)	91.85(2.6-187.6)	
**Propionic acid at 24 months**	n=2	n=41	-
	79.89(18.47-141.32)	86.36(14.58-285.46)	
**Branched butyric acid at 1 month**	n=30	n=10	0.408/-0.13
	2.56(0.19-72.18)	4.61(0.95-30.51)	
**Branched butyric acid at 6 months**	n=26	n=32	0.784/-0.04
	3.19(0.08-14.57)	3.2(1.16-31.6)	
**Branched butyric acid at 12 months**	n=9	n=46	0.609/-0.07
	2.75(0.59-22.54)	5.53(0.67-42.33)	
**Branched butyric acid 24 months**	n=2	n=41	-
	15.27(2.76-27.77)	11.3(1.27-140.25)	
**Linear butyric acid at 1 month**	n=30	n=10	0.767/-0.05
	21.26(2.19-313.88)	35.44(2.74-101.59)	
**Linear butyric acid at 6 months**	n=26	n=32	0.291/-0.14
	30.21(5.35-185.36)	50.07(5.6-222.34)	
**Linear butyric acid at 12 months**	n=9	n=46	0.049/-0.27
	68.34(2.54-123.9)	104.07(4.4-333.58)	
**Linear butyric acid at 24 months**	n=2	n=41	-
	114.11(44.49-183.74)	111.34(14.9-357.45)	
**All butyric acid at 1 month**	n=30	n=10	0.585/-0.09
	26.15(2.37-325.46)	38.56(7.15-106.76)	
**All butyric acid at 6 months**	n=26	n=32	0.277/-0.14
	35.51(5.42-193.54)	52.17(8.97-229.39)	
**All butyric acid at 12 months**	n=9	n=46	0.052/-0.26

	69.79(5.28-124.78)	109.86(5.14-341.71)	
**All butyric acid at 24 months**	n=2	n=41	-
	129.38(47.25-211.5)	129.72(17.17-372.79)	
**All SCFA at 1 month**	n=30	n=10	0.295/-0.17
	266.41(67.32-649.06)	34.02(14.98-621.4)	
**All SCFA at 6 months**	n=26	n=32	0.772/-0.04
	318.69(29.27-601.42)	345.02(73.7-894.84)	
**All SCFA at 12 months**	n=9	n=46	0.055/-0.26
	344.59(67.32-663.59)	444.79(106.55-885.64)	
**All SCFA at 24 months**	n=2	n=41	-
	434.13(353.94-514.32)	447.63(209.65-1163.73)	

p - statistical significance, r - effect size

Table S 17. Effects of type of feeding of children born naturally on SCFA concentrations (μmol/g), median (range).

	Type of feeding		
	Breast feeding	Formula	p/r
Acetic acid at 1 month	n=12	n=4	0.431/-0.20
	159.18(55.12-262.4)	1857.43(135.51-292.8)	
Acetic acid at 6 months	n=10	n=11	0.699/-0.08
	202.59(74.82-348.72)	212.66(41.78-516.45)	
Acetic acid at 12 months	n=5	n=14	0.030/0.50
	165.76(55.12-249)	270.39(150.6-511.2)	
Acetic acid at 24 months	n=2	n=14	-
	224.85(161.5-288.21)	229.18(159.2-388.07)	
Propionic acid at 1 month	n=12	n=4	0.203/-0.32
	25.56(5.07-84.59)	55.36(24.44-106.6)	
Propionic acid at 6 months	n=10	n=11	0.860/0.04
	54.33(6.76-210.69)	51.06(3.16-194.21)	
Propionic acid at 12 months	n=5	n=14	0.105/0.37
	85.1(6.9-128.2)	108.4(2.6-187.6)	
Propionic acid at 24 months	n=2	n=14	-
	79.89(18.46-141.31)	94.93(47.95-179.9)	
Branched butyric acid at 1 month	n=12	n=4	0.671/-0.11
	3.69(0.18-72.18)	4.24(1.3-30.5)	
Branched butyric acid at 6 months	n=10	n=11	0.805/0.05
	4.23(0.07-14.57)	4.19(1.26-11.22)	
Branched butyric acid at 12 months	n=5	n=14	0.890/0.03
	7.37(1.45-22.53)	5.62(0.75-42.33)	
Branched butyric acid at 24 months	n=2	n=14	-
	15.26(2.76-27.77)	11.13(1.87-48.29)	
Linear butyric acid at 1 month	n=12	n=4	0.303/0.26
	11.19(2.18-313.87)	5.08(2.74-8.48)	
Linear butyric acid at 6 months	n=10	n=11	0.549/-0.13
	19.02(5.34-185.35)	42.32(5.6-222.33)	
Linear butyric acid at 12 months	n=5	n=14	0.008/0.61
	29.19(2.54-76.8)	110.29(4.39-264.4)	
Linear butyric acid at 24 months	n=2	n=14	-
	114.11(44.49-183.73)	108.81(29.49-202.3)	
All SCFA at 1 month	n=12	n=4	0.505/-0.17
	229.58(69.47-655.32)	260.34(218.12-423.29)	
All SCFA at 6 months	n=10	n=11	0.751/-0.07
	321.77(167.51-613.4)	391.71(85.79-910.28)	
All SCFA at 12 months	n=5	n=14	0.014/0.56
	319.95(69.47-516.73)	546.34(169.89-912.22)	
All SCFA at 24 months	n=2	n=14	-
	463.52(358.18-568.87)	505.91(322.78-672.02)	

Table S 18. Effects of type of feeding of children born via caesarean section on SCFA concentrations (μmol/g), median (range).

	Type of feeding		
	Breast feeding	Formula	p/r
Acetic acid at 1 month	n=18	n=6	0.368/-0.18
	188.09(96.09-383.74)	257.25(71.9-466.02)	
Acetic acid at 6 months	n=16	n=21	0.571/0.09
	247.04(12.55-435.63)	213.17(62.61-374)	
Acetic acid at 12 months	n=4	n=32	0.940/0.01
	183.5(169.9-388.8)	223.76(84.6-445.09)	
Acetic acid at 24 months	n=0	n=27	-
	0.00(0.00-0.00)	202.99(114.7-686.93)	
Propionic acid at 1 month	n=18	n=6	0.243/-0.24
	29.07(6.48-104.39)	66.95(7.59-91.93)	
Propionic acid at 6 months	n=16	n=21	0.550/-0.10
	54.56(7.04-163.85)	58.58(5.73-180.7)	
Propionic acid at 12 months	n=4	n=32	0.744/0.05
	58.79(49.58-152.6)	64.57(6.09-180.1)	
Propionic acid at 24 months	n=0	n=27	-
	0.00(0.00-0.00)	86.1(14.58-285.45)	
Branched butyric acid at 1 month	n=18	n=6	0.617/-0.10
	2.12(0.26-52.15)	4.8(0.94-20.04)	
Branched butyric acid at 6 months	n=16	n=21	0.530/-0.10
	2.64(0.32-13.97)	2.81(1.15-31.6)	
Branched butyric acid at 12 months	n=4	n=32	0.302/-0.17
	1.49(0.59-21.03)	5.49(0.66-35.19)	
Branched butyric acid at 24 months	n=0	n=27	-
	0.00(0.00-0.00)	11.3(1.26-140.25)	
Linear butyric acid at 1 month	n=18	n=6	0.134/-0.31
	31.63(3.6-209)	59.86(27.93-101.58)	
Linear butyric acid at 6 months	n=16	n=21	0.490/-0.11
	48.02(5.96-140.7)	50.67(6.87-157.39)	
Linear butyric acid at 12 months	n=4	n=32	0.744/-0.05
	103.7(10.59-123.9)	94.34(4.44-333.58)	
Linear butyric acid at 24 months	n=0	n=27	-
	0.00(0.00-0.00)	111.34(14.9-357.45)	
All SCFA at 1 month	n=18	n=6	0.405/-0.17
	302.64(162.8-703.15)	461.35(151.46-631.36)	
All SCFA at 6 months	n=16	n=21	0.842/-0.03
	345.29(33.68-555.93)	362.41(129.47-610.94)	

**All SCFA at 12 months**	n=4	n=32	0.706/-0.06
	358.46(268.03-676.37)	436.18(115.32-695.23)	
**All SCFA at 24 months**	n=0	n=27	-
	0.00(0.00-0.00)	478.21(280.31-1291.24)	

p - statistical significance, r - effect size

Table S 19. Effect of BMI before pregnancy on linear butyric acid concentration.

Stage	Meconium(P1)	7 days(P2)	1 month(P3)	6 months(P4)	12 months(P5)	24 months(P6)
**BMI before pregnancy 18.5-24.99**	n=10	n=12	n=18	n=32	n=28	n=26
**Linear butyric acid(μmol/g) median(range)**	7.2 (1.26-191.3)	11.43 (3.81-195.52)	30.17 (2.54-209)	50.24 (5.6-222.33)	84.03 (2.54-333.58)	113.2 (29.49-357.45)
**BMI before pregnancy <18.5**	n=1	n=5	n=4	n=7	n=6	n=3
**Linear butyric acid(μmol/g) median(range)**	143.92 (143.92-143.92)	6.52 (2.99-8.59)	8.1 (2.73-11.53)	13.59 (8.39-52.3)	95.6 (40.87-218.37)	126.39 (72.95-202.3)
**p, r**	-	0.02(0.55)	0.14(0.32)	0.01(0.40)	0.43(-0.14)	0.97(-0.01)

p - Mann–Whitney test comparing effects of delivery type, r - effect size

Table S 20. Effects of BMI before pregnancy on SCFA concentrations (μmol/g), median (range).

	BMI before pregnancy		
	Normal	Underweight	p/r
Acetic acid at meconium	n=10	n=1	-
	77.08(17.6-328.8)	393.51(393.51-393.51)	
Acetic acid at 7 days	n=12	n=5	0.317/-0.24
	117.41(41.16-372.42)	163.15(123.42-261.47)	
Acetic acid at 1 month	n=18	n=4	0.328/-0.21
	160.26(55.12-342.36)	202.53(166.07-248.74)	
Acetic acid at 6 months	n=32	n=7	0.927/-0.01
	219.63(12.55-516.45)	235.99(62.61-344.99)	
Acetic acid at 12 months	n=28	n=6	0.735/-0.06
	209.53(55.12-511.2)	253.68(104.7-393.51)	
Acetic acid at 24 months	n=26	n=3	0.334/-0.18
	200.89(121.42-686.93)	256.58(168.75-388.07)	
Propionic acid at meconium	n=10	n=1	-
	13.93(1.57-234.5)	89.87(89.87-89.87)	
Propionic acid at 7 days	n=12	n=5	0.635/-0.12
	8.74(4.76-61.74)	18.72(7.83-24.54)	
Propionic acid at 1 month	n=18	n=4	0.328/0.21
	27.64(6.9-106.6)	26.43(6.27-49.9)	
Propionic acid at 6 months	n=32	n=7	0.297/0.17
	65.1(5.73-210.69)	48.57(19.9-94.35)	
Propionic acid at 12 months	n=28	n=6	0.946/0.01
	82.42(6.9-176.68)	78.23(33.81-115.4)	
Propionic acid at 24 months	n=26	n=3	0.163/0.26
	89.65(18.46-212)	47.95(38.32-98.71)	
Branched butyric acid at meconium	n=10	n=1	-
	4.91(0.07-94.75)	10.32(10.32-10.32)	
Branched butyric acid at 7 days	n=12	n=5	0.155/0.35
	1.96(0.4-14.61)	0.48(0.08-35.71)	
Branched butyric acid at 1 month	n=18	n=4	0.469/0.15
	3.1(0.26-72.18)	1.81(0.24-8.9)	
Branched butyric acid at 6 months	n=32	n=7	0.728/0.06
	3.34(1.15-31.6)	3.13(1.65-13.84)	
Branched butyric acid at 12 months	n=28	n=6	0.456/0.13
	6.04(0.79-35.19)	5.46(0.66-10.32)	
Branched butyric acid 24 months	n=26	n=3	0.591/-0.10
	9.71(1.33-31.6)	13.07(12.1-15.24)	
Linear butyric acid at	n=10	n=1	-

**meconium**	7.2(1.26-191.3)	143.92(143.92-143.92)	
**Linear butyric acid at 7 days**	n=12	n=5	0.023/0.55
	11.43(3.81-195.52)	6.52(2.99-8.59)	
**Linear butyric acid at 1 month**	n=18	n=4	0.136/0.32
	30.17(2.54-209)	8.1(2.73-11.53)	
**Linear butyric acid at 6 months**	n=32	n=7	0.014/0.40
	50.24(5.6-222.33)	13.59(8.39-52.37)	
**Linear butyric acid at 12 months**	n=28	n=6	0.429/-0.14
	84.03(2.54-333.58)	95.6(40.87-218.37)	
**Linear butyric acid at 24 months**	n=26	n=3	0.971/-0.01
	113.2(29.49-357.45)	126.39(72.95-202.3)	
**All butyric acid at meconium**	n=10	n=1	-
	12.21(2.36-192.5)	154.25(154.25-154.25)	
**All butyric acid at 7 days**	n=12	n=5	0.082/0.42
	13.55(5.76-210.13)	7.12(3.08-41.4)	
**All butyric acid at 1 month**	n=18	n=4	0.067/0.39
	31.49(4.63-210.86)	9.91(2.97-20.44)	
**All butyric acid at 6 months**	n=32	n=7	0.011/0.41
	59.74(9.68-229.39)	18.56(11.52-55.62)	
**All butyric acid at 12 months**	n=28	n=6	0.456/-0.13
	90.99(5.28-341.71)	98.44(41.68-224.28)	
**All butyric acid at 24 months**	n=26	n=3	0.971/0.01
	135.92(47.25-372.79)	138.49(86.02-217.54)	
**All SCFA at meconium**	n=10	n=1	-
	102.5(22.36-740.77)	637.63(637.63-637.63)	
**All SCFA at 7 days**	n=12	n=5	0.562/-0.14
	139.31(19.64-610.01)	184.78(134.33-293.14)	
**All SCFA at 1 month**	n=18	n=4	0.831/0.05
	254.95(67.32-649.06)	255.74(185.02-275.67)	
**All SCFA at 6 months**	n=32	n=7	0.390/0.14
	349.7(29.27-894.84)	340.54(123.94-467.36)	
**All SCFA at 12 months**	n=28	n=6	0.804/-0.04
	395.25(67.32-867.37)	435.65(212.98-637.63)	
**All SCFA at 24 months**	n=26	n=3	0.914/-0.02
	477.93(283.79-1062.14)	433.41(353.48-653.56)	
	**Normal**	**Overweight**	**p/r**
**Acetic acid at meconium**	n=10	n=5	0.759/0.08

	77.08(17.6-328.8)	90.97(12.42-166.5)	
**Acetic acid at 7 days**	n=12	n=8	0.847/-0.04
	117.41(4.11-372.42)	141.72(32.24-430.51)	
**Acetic acid at 1 month**	n=18	n=9	0.368/-0.17
	160.26(55.12-342.36)	250.84(113.5-383.74)	
**Acetic acid at 6 months**	n=32	n=11	0.568/0.09
	219.63(12.55-516.45)	208.5(105.12-305.93)	
**Acetic acid at 12 months**	n=28	n=14	0.386/-0.13
	209.53(55.12-511.2)	247.18(84.6-393.51)	
**Acetic acid at 24 months**	n=26	n=7	0.140/-0.26
	200.89(121.42-686.93)	239.01(164.96-613.82)	
**Propionic acid at meconium**	n=10	n=5	0.854/0.05
	13.93(1.57-234.5)	12.17(2.37-46.28)	
**Propionic acid at 7 days**	n=12	n=8	0.616/-0.11
	8.74(4.76-61.74)	15.04(5.07-52.7)	
**Propionic acid at 1 month**	n=18	n=9	0.589/0.10
	27.64(6.9-106.6)	24.44(5.07-91.93)	
**Propionic acid at 6 months**	n=32	n=11	0.136/0.23
	65.1(5.73-210.69)	32.1(3.16-194.21)	
**Propionic acid at 12 months**	n=28	n=14	0.431/-0.12
	82.42(6.9-176.68)	96.3(6.09-180.1)	
**Propionic acid at 24 months**	n=26	n=7	0.261/-0.20
	89.65(18.46-212)	116.4(60.46-285.45)	
**Branched butyric acid at meconium**	n=10	n=5	0.854/-0.05
	4.91(0.07-94.75)	6.74(0.5-9.83)	
**Branched butyric acid at 7 days**	n=12	n=8	0.787/-0.06
	1.96(0.4-14.61)	1.89(0.55-5.25)	
**Branched butyric acid at 1 month**	n=18	n=9	0.817/0.04
	3.1(0.26-72.18)	1.87(0.18-52.15)	
**Branched butyric acid at 6 months**	n=32	n=11	0.168/0.21
	3.34(1.15-31.6)	1.66(0.07-11.47)	
**Branched butyric acid at 12 months**	n=28	n=14	0.779/-0.04
	6.04(0.79-35.19)	5.76(0.59-42.33)	
**Branched butyric acid 24 months**	n=26	n=7	0.982/0.00
	9.71(1.33-31.6)	9.72(1.92-28.67)	
**Linear butyric acid at meconium**	n=10	n=5	0.951/-0.02
	7.2(1.26-191.3)	10.07(1.19-28.39)	
**Linear butyric acid at 7 days**	n=12	n=8	0.847/-0.04
	11.43(3.81-195.52)	11.76(5.99-59.59)	
**Linear butyric acid at 1 month**	n=18	n=9	0.190/0.25
	30.17(2.54-209)	5.29(2.18-156.47)	
**Linear butyric acid at 6 months**	n=32	n=11	0.004/0.44
	50.24(5.6-222.33)	16.38(5.34-82.56)	
**Linear butyric acid at 12**	n=28	n=14	0.146/-

**months**	84.03(2.54-333.58)	122.75(6.29-236.87)	0.22
**Linear butyric acid at 24 months**	n=26<br>113.2(29.49-357.45)	n=7<br>134.6(44.34-235.77)	0.582/0.10
**All butyric acid at meconium**	n=10<br>12.21(2.36-192.5)	n=5<br>16.81(1.7-36.29)	0.951/0.02
**All butyric acid at 7 days**	n=12<br>13.55(5.76-210.13)	n=8<br>16.3(7.28-61.34)	0.616/-0.11
**All butyric acid at 1 month**	n=18<br>31.49(4.63-210.86)	n=9<br>33.25(2.37-170.41)	0.662/0.08
**All butyric acid at 6 months**	n=32<br>59.74(9.68-229.39)	n=11<br>17.37(5.42-94.04)	0.002/0.48
**All butyric acid at 12 months**	n=28<br>90.99(5.28-341.71)	n=14<br>130.37(15.86-239.63)	0.112/-0.24
**All butyric acid at 24 months**	n=26<br>135.92(47.25-372.79)	n=7<br>138.62(54.06-264.44)	0.613/0.09
**All SCFA at meconium**	n=10<br>102.5(22.36-740.77)	n=5<br>126.81(16.5-247.14)	0.759/0.08
**All SCFA at 7 days**	n=12<br>139.31(19.64-610.01)	n=8<br>186.36(54.97-507.52)	0.728/-0.08
**All SCFA at 1 month**	n=18<br>254.95(67.32-649.06)	n=9<br>258.29(139.93-492.21)	0.625/-0.09
**All SCFA at 6 months**	n=32<br>349.7(29.27-894.84)	n=11<br>280.97(125.09-488.15)	0.152/0.22
**All SCFA at 12 months**	n=28<br>395.25(67.32-867.37)	n=14<br>518.04(106.55-663.59)	0.317/-0.15
**All SCFA at 24 months**	n=26<br>477.93(283.79-1062.14)	n=7<br>498.42(312.87-1163.73)	0.552/-0.10
	**Normal**	**Obesity**	**p/r**
**Acetic acid at meconium**	n=10<br>77.08(17.6-328.8)	n=3<br>96.69(67.33-259.36)	0.673/-0.12
**Acetic acid at 7 days**	n=12<br>117.41(4.11-372.42)	n=5<br>129.26(69.61-264.67)	0.874/0.04
**Acetic acid at 1 month**	n=18<br>160.26(55.12-342.36)	n=8<br>199.24(71.9-466.02)	0.598/-0.10
**Acetic acid at 6 months**	n=32<br>219.63(12.55-516.45)	n=6<br>252.41(169.2-345.95)	0.617/-0.08
**Acetic acid at 12 months**	n=28<br>209.53(55.12-511.2)	n=5<br>249(182.3-428.3)	0.259/-0.20
**Acetic acid at 24 months**	n=26<br>200.89(121.42-686.93)	n=7<br>212.5(122.18-233.39)	0.582/0.10

**Propionic acid at meconium**	n=10	n=3	0.800/-0.07
	13.93(1.57-234.5)	17.58(7.83-73.97)	
**Propionic acid at 7 days**	n=12	n=5	0.188/-0.32
	8.74(4.76-61.74)	16.27(13.72-52.27)	
**Propionic acid at 1 month**	n=18	n=8	0.331/-0.19
	27.64(6.9-106.6)	60.62(10.7-84.59)	
**Propionic acid at 6 months**	n=32	n=6	0.645/0.07
	65.1(5.73-210.69)	49.32(29.42-180.7)	
**Propionic acid at 12 months**	n=28	n=5	0.530/-0.11
	82.42(6.9-176.68)	68.03(45.51-187.6)	
**Propionic acid at 24 months**	n=26	n=7	0.390/0.15
	89.65(18.46-212)	75.14(14.58-169.7)	
**Branched butyric acid at meconium**	n=10	n=3	0.933/0.02
	4.91(0.07-94.75)	1.9(0.24-15.7)	
**Branched butyric acid at 7 days**	n=12	n=5	0.082/-0.42
	1.96(0.4-14.61)	6.46(1.55-17.24)	
**Branched butyric acid at 1 month**	n=18	n=8	0.487/-0.14
	3.1(0.26-72.18)	4.8 (0.74-20.04)	
**Branched butyric acid at 6 months**	n=32	n=6	0.271/-0.18
	3.34(1.15-31.6)	6.16(1.58-20.66)	
**Branched butyric acid at 12 months**	n=28	n=5	0.900/-0.02
	6.04(0.79-35.19)	5.34(1.45-21.03)	
**Branched butyric acid 24 months**	n=26	n=7	0.676/-0.07
	9.71(1.33-31.6)	13.95(1.26-140.25)	
**Linear butyric acid at meconium**	n=10	n=3	0.272/-0.30
	7.2(1.26-191.3)	13.99(9.3-84.01)	
**Linear butyric acid at 7 days**	n=12	n=5	0.958/0.01
	11.43(3.81-195.52)	15.18(6.48-43.68)	
**Linear butyric acid at 1 month**	n=18	n=8	0.232/-0.23
	30.17(2.54-209)	48.69(11.15-313.87)	
**Linear butyric acid at 6 months**	n=32	n=6	0.459/-0.12
	50.24(5.6-222.33)	77(16.92-185.35)	
**Linear butyric acid at 12 months**	n=28	n=5	0.380/-0.15
	84.03(2.54-333.58)	85.8(48.76-264.4)	
**Linear butyric acid at 24 months**	n=26	n=7	0.322/0.17
	113.2(29.49-357.45)	97.93(45.03-176.35)	
**All butyric acid at meconium**	n=10	n=3	0.933/-0.02
	12.21(2.36-192.5)	15.9(9.55-99.72)	
**All butyric acid at 7 days**	n=12	n=5	0.874/-0.04
	13.56(5.77-210.13)	21.65(9.48-51.7)	
**All butyric acid at 1 month**	n=18	n=8	0.304/-0.20
	31.5(46.37-210.87)	59.49(14.09-325.47)	
**All butyric acid at 6 months**	n=32	n=6	0.368/-0.15
	59.74(9.68-229.39)	88.13(20-193.55)	
**All butyric acid at 12 months**	n=28	n=5	0.303/-

	90.99(5.29-341.72)	106.84(58.3-269.74)	0.18
**All butyric acid at 24 months**	n=26	n=7	0.809/0.04
	135.93(47.26-372.79)	129.72(54.35-224.65)	
**All SCFA at meconium**	n=10	n=3	0.800/-0.07
	102.51(22.36-740.77)	130.18(84.71-433)	
**All SCFA at 7 days**	n=12	n=5	0.874/0.04
	139.31(19.64-610.02)	201.28(94.15-366.5)	
**All SCFA at 1 month**	n=18	n=8	0.331/-0.19
	254.95(67.32-649.0.7)	353.73(149.83-632.65)	
**All SCFA at 6 months**	n=32	n=6	0.617/-0.08
	349.71(29.28-894.85)	381(225.26-601.42)	
**All SCFA at 12 months**	n=28	n=5	0.407/-0.14
	395.25(67.32-867.37)	447(356.82-885.64)	
**All SCFA at 24 months**	n=26	n=7	0.209/0.22
	477.94(283.79-1062.14)	428.4(270.29-566.28)	

p - statistical significance, r - effect size

Table S 21. Effect of weight gain during pregnancy on branched chain butyric acid concentration.

Stage	**Meconium(P1)**	**7 days(P2)**	**1 month(P3)**	**6 months(P4)**	**12 months(P5)**	**24 months(P6)**
Inadequate	n=3	n=7	n=9	n=10	n=9	n=12
Branched butyric acid(μmol/g) median(range)	10.32 (0.1-15.7)	1.97 (0.11-35.71)	2.31 (0.24-30.5)	3.08 (1.3-20.66)	5.91 (0.98-42.33)	6.74 (1.26-26.4)
Adequate	n=4	n=4	n=8	n=12	n=10	n=6
Branched butyric acid(μmol/g) median(range)	6.75 (0.07-15.18)	1.13 (0.43-4.02)	3.1 (0.26-8.99)	3.84 (1.24-13.97)	7.31 (0.8-22.53)	24.77 (10.21-31.6)
Excessive	n=11	n=17	n=21	n=33	n=33	n=25
Branched butyric acid(μmol/g) median(range)	5.49 (0.24-94.75)	2.21 (0.4-17.24)	1.87 (0.18-72.18)	3.01 (0.07-31.6)	5.26 (0.59-35.19)	11.3 (1.33-140.25)
p, r	0.78(0.03)	0.4(0.07)	0.96(0.002)	0.31(0.04)	0.59(0.02)	0.02(0.19)

p - Mann–Whitney test comparing effects of weight gain, r - effect size

Table S 22.Effect of weight gain during pregnancy on all butyrate content.

Stage	Meconium(P1)	7 days(P2)	1 month(P3)	6 months(P4)	12 months(P5)	24 months(P6)
**Inadequate**	n=3	n=7	n=9	n=10	n=9	n=12
**All butyric acid(μmol/g) median(range)**	99.71 (2.79-154.25)	11.43 (5.76-146.08)	29.48 (2.97-121.92)	45.88 (8.97-142.67)	126.18 (5.28-332.39)	86.41 (47.25-162.37)
**Adequate**	n=4	n=4	n=8	n=12	n=10	n=6
**All butyric acid(μmol/g) median(range)**	17.96 (5.1-23.65)	13.79 (9.48-25.31)	25.57 (4.63-99.42)	53.92 (13.14-104.91)	74.77 (36.42-269.74)	220.66 (76.15-372.79)
**Excessive**	n=11	n=17	n=21	n=33	n=33	n=25
**All butyric acid(μmol/g) median(range)**	12.11 (1.7-192.5)	16.57 (7.14-210.13)	26.98 (2.37-325.46)	39.84 (5.42-229.39)	110.4 (8.66-341.71)	139.42 (54.06-330.2)
**p, r**	0.73(0.04)	0.79(0.02)	0.63(0.01)	0.88(0.01)	0.8(0.01)	0.02(0.2)

p - Mann–Whitney test comparing effects of weight gain, r - effect size

Table S 23. Effects of weight gain during pregnancy on SCFA concentrations (μmol/g), median (range) .

	Weight gain during pregnancy			
	**Inadequate**	**Adequate**	**Excessive**	**p/r**
**Acetic acid at meconium**	n=3	n=4	n=11	0.67/0.047
	259.36(17.6-393.51)	64.93(32.46-320.53)	86(12.42-258.8)	
**Acetic acid at 7 days**	n=7	n=4	n=17	0.9/0.008
	123.99(4.11-430.51)	133.4(70.95-163.84)	137.9(32.24-372.42)	
**Acetic acid at 1 month**	n=9	n=8	n=21	0.71/0.019
	166.07(71.9-466.02)	169.83(55.12-342.36)	214.03(110.4-383.74)	
**Acetic acid at 6 months**	n=10	n=12	n=33	0.98/0.006
	212.91(169.2-348.72)	228.57(41.78-374)	228.29(12.55-516.45)	
**Acetic acid at 12 months**	n=9	n=10	n=33	0.73/0.012
	249(55.12-394.78)	213.93(125.65-511.2)	224.23(84.6-445.09)	
**Acetic acid at 24 months**	n=12	n=6	n=25	0.88/0.006
	203.8(140.8-294.35)	199.56(161.5-243.8)	228.53(121.42-686.93)	
**Propionic acid at meconium**	n=3	n=4	n=11	0.86/0.017
	73.97(1.96-89.87)	12.19(2-164.53)	14.97(1.57-117.6)	
**Propionic acid at 7 days**	n=7	n=4	n=17	0.28/0.094
	16.27(5.57-33.2)	24.62(13.72-61.74)	10.98(4.76-52.7)	
**Propionic acid at 1 month**	n=9	n=8	n=21	0.75/0.015
	48.44(6.27-106.6)	23.98(6.9-91.93)	30.68(5.07-104.39)	
**Propionic acid at 6 months**	n=10	n=12	n=33	0.91/0.003
	68.66(3.16-210.69)	49.6(9.12-194.21)	54.7(5.73-180.7)	
**Propionic acid at 12 months**	n=9	n=10	n=33	0.08/0.098
	89.87(6.9-148.06)	100.19(15.38-187.6)	62.54(6.09-180.1)	
**Propionic acid at 24 months**	n=12	n=6	n=25	0.11/0.106
	78.59(18.46-116.4)	95.25(66.59-150.62)	98.96(14.58-285.45)	
**Branched butyric acid at meconium**	n=3	n=4	n=11	0.78/0.029
	10.32(0.1-15.7)	6.75(0.07-15.18)	5.49(0.24-94.75)	
**Branched butyric acid at 7 days**	n=7	n=4	n=17	0.4/0.067
	1.97(0.11-35.71)	1.13(0.43-4.02)	2.21(0.4-17.24)	
**Branched butyric acid at**	n=9	n=8	n=21	0.96/0.002

**1 month**	2.31(0.24-30.5)	3.1(0.26-8.99)	1.87(0.18-72.18)	
**Branched butyric acid at 6 months**	n=10	n=12	n=33	0.31/0.044
	3.08(1.3-20.66)	3.84(1.24-13.97)	3.01(0.07-31.6)	
**Branched butyric acid at 12 months**	n=9	n=10	n=33	0.59/0.021
	5.91(0.98-42.33)	7.31(0.8-22.53)	5.26(0.59-35.19)	
**Branched butyric acid 24 months**	n=12	n=6	n=25	0.02/0.185
	6.74(1.26-26.4)	24.77(10.21-31.6)	11.3(1.33-140.25)	
**Linear butyric acid at meconium**	n=3	n=4	n=11	0.66/0.049
	84.01(2.69-143.92)	8.53(5.02-13.82)	9.3(1.19-191.3)	
**Linear butyric acid at 7 days**	n=7	n=4	n=17	0.32/0.084
	6.52(3.81-134.24)	11.56(7.93-24.7)	11.83(5.94-195.52)	
**Linear butyric acid at 1 month**	n=9	n=8	n=21	0.7/0.019
	12.5(2.73-116.05)	22.83(2.54-90.43)	22.95(2.18-31.387)	
**Linear butyric acid at 6 months**	n=10	n=12	n=33	0.89/0.004
	34.57(7.67-140.7)	49.81(5.6-97.66)	38.21(5.34-222.33)	
**Linear butyric acid at 12 months**	n=9	n=10	n=33	0.78/0.01
	96.77(2.54-329.04)	72.67(25.23-264.4)	105.13(4.44-333.58)	
**Linear butyric acid at 24 months**	n=12	n=6	n=25	0.05/0.146
	80.98(29.49-160.5)	191.43(54.39-357.45)	134.6(44.34-314.01)	
**All butyric acid at meconium**	n=3	n=4	n=11	0.73/0.037
	99.71(2.79-154.25)	17.96(5.1-23.65)	12.11(1.7-192.5)	
**All butyric acid at 7 days**	n=7	n=4	n=17	0.79/0.018
	11.42(5.76-146.08)	13.79(9.48-25.41)	16.57(7.14-210.13)	
**All butyric acid at 1 month**	n=9	n=8	n=21	0.63/0.025
	29.48(2.97-121.92)	25.57(4.63-99.42)	26.98(2.37-325.46)	
**All butyric acid at 6 months**	n=10	n=12	n=33	0.88/0.005
	45.88(8.97-142.67)	53.92(13.14-104.91)	39.84(5.42-229.39)	
**All butyric acid at 12 months**	n=9	n=10	n=33	0.8/0.009
	126.18(5.28-332.39)	74.77(36.42-269.74)	110.4(8.66-341.71)	
**All butyric acid at 24 months**	n=12	n=6	n=25	0.02/0.198
	86.41(47.25-162.37)	220.66(76.15-372.79)	139.42(54.06-330.24)	
**All SCFA at meconium**	n=3	n=4	n=11	0.74/0.036
	433.05(22.36-	97.56(45.99-	113.09(16.5-	

	637.63)	497.37)	568.9)	
**All SCFA at 7 days**	n=7	n=4	n=17	0.89/0.009
	184.78(19.64-507.52)	181.49(94.15-231.66)	183.21(54.97-610.01)	
**All SCFA at 1 month**	n=9	n=8	n=21	0.42/0.046
	214.55(149.82-621.4)	217.44(67.32-475.85)	284.36(139.93-649.06)	
**All SCFA at 6 months**	n=10	n=12	n=33	0.96/0.001
	340.92(199.34-595.13)	333.24(73.7-551.35)	329.09(29.27-894.84)	
**All SCFA at 12 months**	n=9	n=10	n=33	0.66/0.016
	523.65(67.32-637.63)	388.26(188.92-885.64)	434.71(106.55-687.87)	
**All SCFA at 24 months**	n=12	n=6	n=25	0.02/0.178
	360.54(270.29-520.16)	527.02(401.27-633.66)	482.05(294.96-1163.73)	

p - statistical significance, r - effect size

Table S 24. Effects of children body mass on SCFA concentrations (μmol/g), median (range).

	Body mass		
	≥85th percentile	>15th - <85th percentile	p/r
**Acetic acid at 1 month**	n=2	n=37	-
	201.9(141.39-262.4)	182.21(55.12-466.02)	
**Acetic acid at 6 months**	n=7	n=49	0.442/0.10
	240.98(107.2-338.2)	212.66(12.55-516.45)	
**Acetic acid at 12 months**	n=7	n=48	0.605/0.07
	234(205-263)	223.76(55.12-445.09)	
**Acetic acid at 24 months**	n=5	n=40	0.459/-0.11
	182.25(122.18-290.29)	208.15(114.7-686.93)	
**Propionic acid at 1 month**	n=2	n=37	-
	89.66(74.94-104.39)	27.82(5.07-100.9)	
**Propionic acid at 6 months**	n=7	n=49	0.442/0.10
	57.05(30.7-138.6)	53.44(3.16-210.69)	
**Propionic acid at 12 months**	n=7	n=48	0.950/-0.01
	69.31(2.6-153.4)	89.87(6.09-187.6)	
**Propionic acid at 24 months**	n=5	n=40	0.928/0.01
	98.96(22.32-169.7)	86.23(14.58-285.45)	
**Branched butyric acid at 1 month**	n=2	n=37	-
	14.57(4.64-24.51)	2.37(0.18-72.18)	
**Branched butyric acid at 6 months**	n=7	n=49	0.346/-0.13
	2.27(1.26-11.47)	3.25(0.07-31.6)	
**Branched butyric acid at 12 months**	n=7	n=48	0.520/-0.09
	5.26(0.75-9.53)	5.81(0.59-42.33)	
**Branched butyric acid 24 months**	n=5	n=40	0.187/-0.20
	2.15(1.26-22.36)	11.7(1.33-140.25)	
**Branched butyric acid at 1 month**	n=2	n=37	-
	0.7(0.4-7.0)	2.5(0.4-32.7)	
**Linear butyric acid at 1 month**	n=2	n=37	-
	75.07(23.75-126.39)	22.95(2.18-313.87)	
**Linear butyric acid at 6 months**	n=7	n=49	0.037/0.28
	68.29(27.21-128.4)	38.21(5.34-222.33)	
**Linear butyric acid at 12 months**	n=7	n=48	0.605/-0.07
	96.77(4.39-195.1)	92.76(2.54-333.58)	
**Linear butyric acid at 24 months**	n=5	n=40	0.551/-0.09
	97.93(63.69-141.38)	113.2(14.9-357.45)	
**All butyric acid at 1 month**	n=2	n=37	-
	89.65(28.39-150.9)	29.48(2.37-325.46)	
**All butyric acid at 6 months**	n=7	n=49	0.047/0.26
	70.57(30.22-129.66)	45.24(5.42-229.39)	

**All butyric acid at 12 months**	n=7	n=48	0.487/-0.09
	105.09(5.14-202.77)	108.07(5.28-341.71)	
**All butyric acid at 24 months**	n=5	n=40	0.416/-0.12
	99.85(65.84-154.05)	138.56(17.17-372.79)	
**All SCFA at 1 month**	n=2	n=37	-
	381.21(365.73-396.69)	258.29(67.32-649.06)	
**All SCFA at 6 months miesiąc**	n=7	n=49	0.334/0.13
	418.91(186.75-530.02)	326.06(29.27-894.84)	
**All SCFA at 12 months**	n=7	n=48	0.910/-0.02
	420.49(158.34-867.37)	435.65(67.32-885.64)	
**All SCFA at 24 months**	n=5	n=40	0.240/-0.17
	334.3(294.96-543.31)	452.18(209.65-1163.73)	

p - statistical significance, r - effect size

Table S 25. Effect of birth weight on SCFA concentrations (μmol/g), median (range).

	Birth weight		
	**≤15th percentile**	**≥85th percentile**	**p/r**
**Acetic acid at meconium**	n=1	n=3	-
	328.8.(328.8-328.8)	86(17.6-258.8)	
**Acetic acid at 7 days**	n=2	n=6	-
	63.77(4.12-123.42)	120.9(75.27-372.42)	
**Acetic acid at 1 month**	n=4	n=7	0.299/-0.31
	218.77(71.9-292.8)	262.4(141.4-383.75)	
**Acetic acid at 6 months**	n=6	n=9	0.377/0.23
	255.4(107.2-360.8)	200.3(12.56-345.95)	
**Acetic acid at 12 months**	n=5	n=8	0.341/-0.26
	176.7(55.13-511.2)	237.22(150.6-382.87)	
**Acetic acid at 24 months**	n=6	n=7	0.074/-0.50
	197.06(169.6-233.37)	288.22(140.6-613.83)	
**Propionic acid at meconium**	n=1	n=3	-
	234.5(234.5-234.5)	14.97(1.97-117.6)	
**Propionic acid at 7 days**	n=2	n=6	-
	6.7(5.57-7.83)	7.7(4.76-44.52)	
**Propionic acid at 1 month**	n=4	n=7	0.925/-0.03
	57.97(46.38-106.6)	84.32(23.9-104.39)	
**Propionic acid at 6 months**	n=6	n=9	0.860/0.05
	63.45(16-210.7)	61.93(7.04-145.2)	
**Propionic acid at 12 months**	n=5	n=8	0.272/0.30
	62.54(6.9-153.4)	46.36(2.6-85.1)	
**Propionic acid at 24 months**	n=6	n=7	0.432/-0.22
	82.92(22.33-150.63)	108.24(18.47-285.46)	
**Branched butyric acid at meconium**	n=1	n=3	-
	20.64(20.64-20.64)	1.2(0.1-6.12)	
**Branched butyric acid at 7 days**	n=2	n=6	-
	1.03(0.09-1.97)	2.56(1.2-14.61)	
**Branched butyric acid at 1 month**	n=4	n=7	0.777/-0.09
	3.28(1.3-5.88)	4.64(0.57-24.51)	
**Branched butyric acid at 6 months**	n=6	n=9	0.953/0.02
	3.6(1.98-14.57)	4.01(1.26-11.47)	
**Branched butyric acid at 12 months**	n=5	n=8	0.942/-0.02
	2.75(0.99-7.67)	3.79(0.75-7.72)	

**Branched butyric acid at 24 months**	n=6	n=7	0.284/-0.30
	2.19(1.27-31.6)	12.67(1.7-48.3)	
**Linear butyric acid at meconium**	n=1	n=3	-
	156.82(156.82-156.82)	5.97(2.69-191.3)	
**Linear butyric acid at 7 days**	n=2	n=6	-
	5.49(3-7.99)	22.71(3.82-195.52)	
**Linear butyric acid at 1 month**	n=4	n=7	0.299/-0.31
	18.21(5.86-116.05)	101.36(7.77-313.88)	
**Linear butyric acid at 6 months**	n=6	n=9	0.860/-0.05
	34.77(12.44-140.7)	68.17(6.87-185.36)	
**Linear butyric acid at 12 months**	n=5	n=8	0.510/-0.18
	125.2(2.54-195.1)	137.7(4.4-242.73)	
**Linear butyric acid at 24 months**	n=6	n=7	0.225/-0.34
	99.44(29.49-160.5)	141.38(44.49-271.49)	
**All SCFA at meconium**	n=1	n=3	-
	775.04(775.04-775.04)	121.72(27.13-600.76)	
**All SCFA at 7 days**	n=2	n=6	-
	88.6(32.72-144.49)	190.15(104.31-621.26)	
**All SCFA at 1 month**	n=4	n=7	0.156/-0.43
	352.08(151.47-431.2)	446.99(345-703.16)	
**All SCFA at 6 months**	n=6	n=9	0.596/0.14
	453.84(204.28-601.7)	375.78(33.68-613.4)	
**All SCFA at 12 months**	n=5	n=8	0.510/-0.18
	389.27(69.47-887.9)	434.91(169.9-632.96)	
**All SCFA at 24 months**	n=6	n=7	0.175/-0.38
	439.71(338.21-509.8)	557.58(358.18-1291.25)	

p - statistical significance, r - effect size

Table S 26. Effects of sex on SCFA concentrations (μmol/g), median (range).

	Sex		
	**Female**	**Male**	**p/r**
**Acetic acid at meconium**	n=11	n=9	0.261/-0.25
	67.33(12.42-328.8)	96.69(32.46-393.51)	
**Acetic acid at 7 days**	n=14	n=17	0.186/-0.24
	80.66(32.24-430.51)	144.5(4.11-264.67)	
**Acetic acid at 1 month**	n=19	n=22	0.948/-0.01
	175.9(55.12-466.02)	186.62(72.87-337.3)	
**Acetic acid at 6 months**	n=24	n=35	0.765/-0.04
	203.24(74.82-435.63)	226.1(12.55-516.45)	
**Acetic acid at 12 months**	n=26	n=30	0.851/-0.02
	227.25(84.6-445.09)	233.6(55.12-511.2)	
**Acetic acid at 24 months**	n=23	n=22	0.039/0.31
	225(114.7-686.93)	187.01(121.42-304.82)	
**Propionic acid at meconium**	n=11	n=9	0.370/-0.20
	7.83(1.96-234.5)	16.77(1.57-164.53)	
**Propionic acid at 7 days**	n=14	n=17	0.984/0.00
	14.69(5.1-52.7)	14.71(4.76-61.74)	
**Propionic acid at 1 month**	n=19	n=22	0.560/-0.09
	30.68(5.07-106.6)	33.24(6.27-104.39)	
**Propionic acid at 6 months**	n=24	n=35	0.403/-0.11
	52.74(3.16-180.7)	61.21(5.73-210.69)	
**Propionic acid at 12 months**	n=26	n=30	0.400/0.11
	91.85(6.09-187.6)	73.88(2.6-176.68)	
**Propionic acid at 24 months**	n=23	n=22	0.727/-0.05
	86.36(18.46-285.45)	89.53(14.58-212)	
**Branched butyric acid at meconium**	n=11	n=9	0.295/-0.24
	5.49(0.07-20.64)	7.61(1.09-94.75)	
**Branched butyric acid at 7 days**	n=14	n=17	0.246/0.21
	2.55(0.14-14.61)	1.2(0.08-35.71)	
**Branched butyric acid at 1 month**	n=19	n=22	0.097/-0.26
	1.58(0.18-30.5)	4.92(0.24-72.18)	
**Branched butyric acid at 6 months**	n=24	n=35	0.345/-0.12
	2.96(0.32-31.6)	4.01(0.07-15.47)	
**Branched butyric acid at 12 months**	n=26	n=30	0.877/0.02
	5.52(0.59-42.33)	5.51(0.66-35.19)	
**Branched butyric acid 24 months**	n=23	n=22	0.087/-0.26
	6.75(1.26-48.29)	14.11(1.33-140.25)	
**Linear butyric acid at meconium**	n=11	n=9	0.456/-0.17
	5.97(1.19-156.82)	13.82(1.26-191.3)	
**Linear butyric acid at 7 days**	n=14	n=17	0.444/0.14
	11.63(1.47-195.52)	9.99(2.99-60.27)	

**Linear butyric acid at 1 month**	n=19	n=22	0.385/-0.14
	12.5(2.18-313.87)	30.39(2.73-209)	
**Linear butyric acid at 6 months**	n=24	n=35	0.812/0.03
	48.11(7.67-185.35)	45.35(5.34-222.33)	
**Linear butyric acid at 12 months**	n=26	n=30	0.678/0.05
	98.96(4.44-329.04)	93.03(2.54-333.58)	
**Linear butyric acid at 24 months**	n=23	n=22	0.105/0.24
	141.38(14.9-314.01)	93.02(29.49-357.45)	
**All butyric acid at meconium**	n=11	n=9	0.201/-0.29
	11.13(1.7-177.46)	23.62(2.36-192.5)	
**All butyric acid at 7 days**	n=14	n=17	0.399/0.15
	15.08(1.61-210.13)	11.61(3.08-60.67)	
**All butyric acid at 1 month**	n=19	n=22	0.270/-0.17
	24.36(2.37-325.46)	42.08(2.97-210.86)	
**All butyric acid at 6 months**	n=24	n=35	0.982/0.00
	49.62(8.97-193.54)	47.71(5.42-229.39)	
**All butyric acid at 12 months**	n=26	n=30	0.619/0.07
	109.86(12.68-332.39)	105.45(5.14-341.71)	
**All butyric acid at 24 months**	n=23	n=22	0.316/0.15
	154.05(17.17-330.24)	116.85(55.89-372.79)	
**All SCFA at meconium**	n=11	n=9	0.230/-0.27
	88.99(16.5-740.77)	130.17(38.59-637.63)	
**All SCFA at 7 days**	n=14	n=17	0.297/-0.19
	115.39(54.97-610.01)	194.35(19.64-366.5)	
**All SCFA at 1 month**	n=19	n=22	0.727/-0.06
	258.29(67.32-632.64)	277.99(131.81-649.06)	
**All SCFA at 6 months**	n=24	n=35	0.640/-0.06
	311.9(183.06-601.42)	348.74(292.79-894.84)	
**All SCFA at 12 months**	n=26	n=30	0.702/0.05
	427.6(106.55-885.64)	439.34(67.32-867.37)	
**All SCFA at 24 months**	n=23	n=22	0.121/0.23
	498.42(209.65-1163.73)	414.83(283.79-747.44)	

p - statistical significance, r - effect size

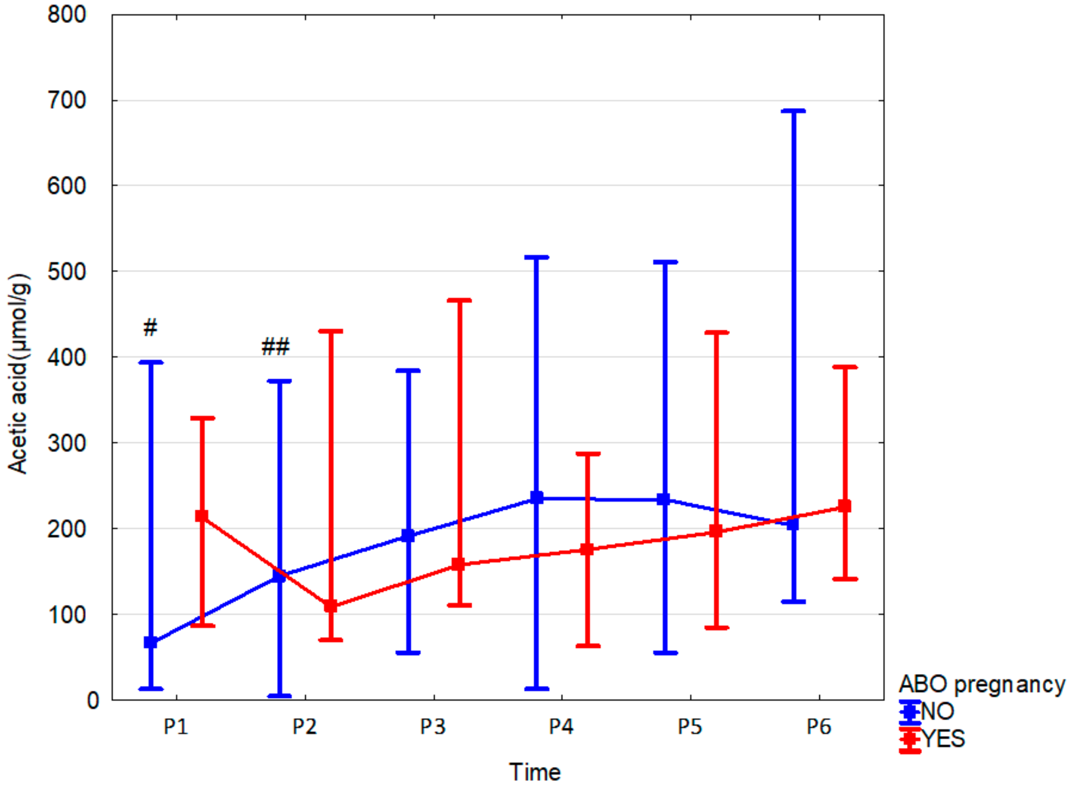

Figure S 1. Acetic acid concentrations (median) in children over time of mothers with (red line) or without (blue line) antibiotic therapy during pregnancy (ABO).

Legend: Error bars represent range. Wilcoxon paired tests regarding time (# No ABO, $ ABO): # $p < 0.05$ P1 vs P2,P3,P4,P5 and P6; ##: $p < 0.05$ P2 vs P3,P4,P5 and P6.

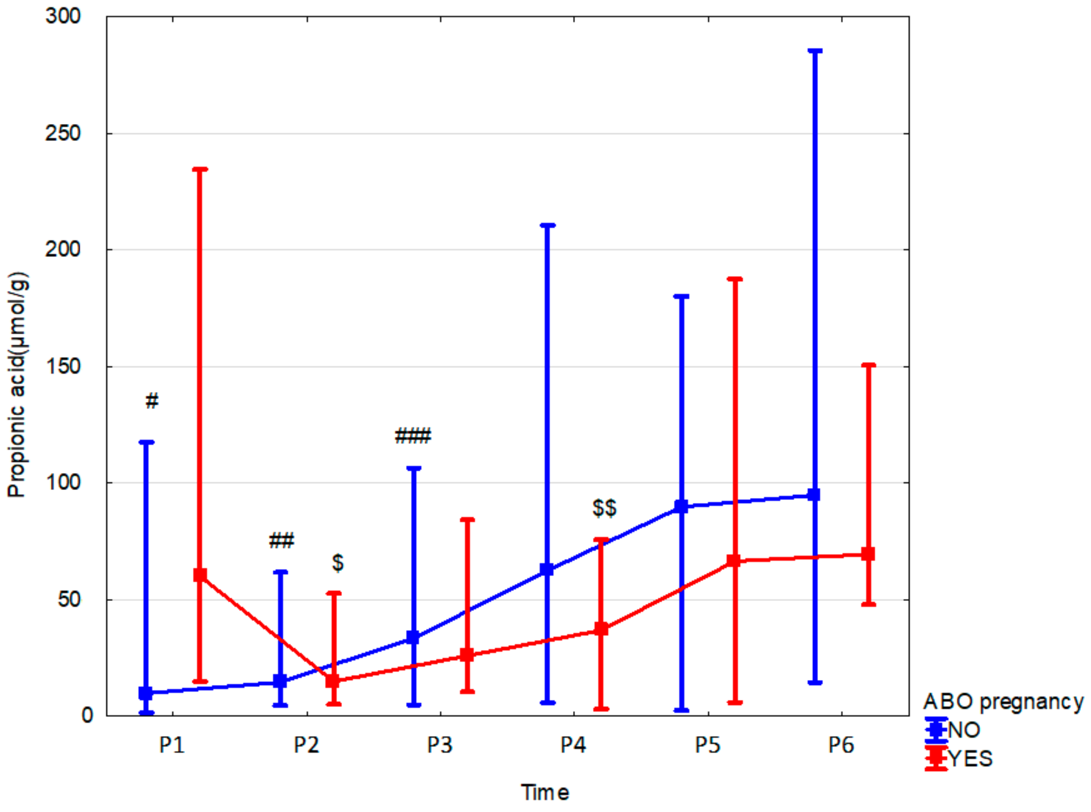

Figure S 2. Propionic acid concentrations (median) in children over time of mothers with (red line) or without (blue line) antibiotic therapy during pregnancy (ABO).

Legend: Error bars represent range. Wilcoxon paired tests regarding time: (# No ABO, $ ABO): # $p < 0.05$ P1 vs P2,P4,P5 and P6; ##,$: $p < 0.05$ P2 vs P4,P5 and P6; ### $p<0.05$ P3 vs P4,P5 and P6; $$ $p<0.05$ P4 vs P5 and P6

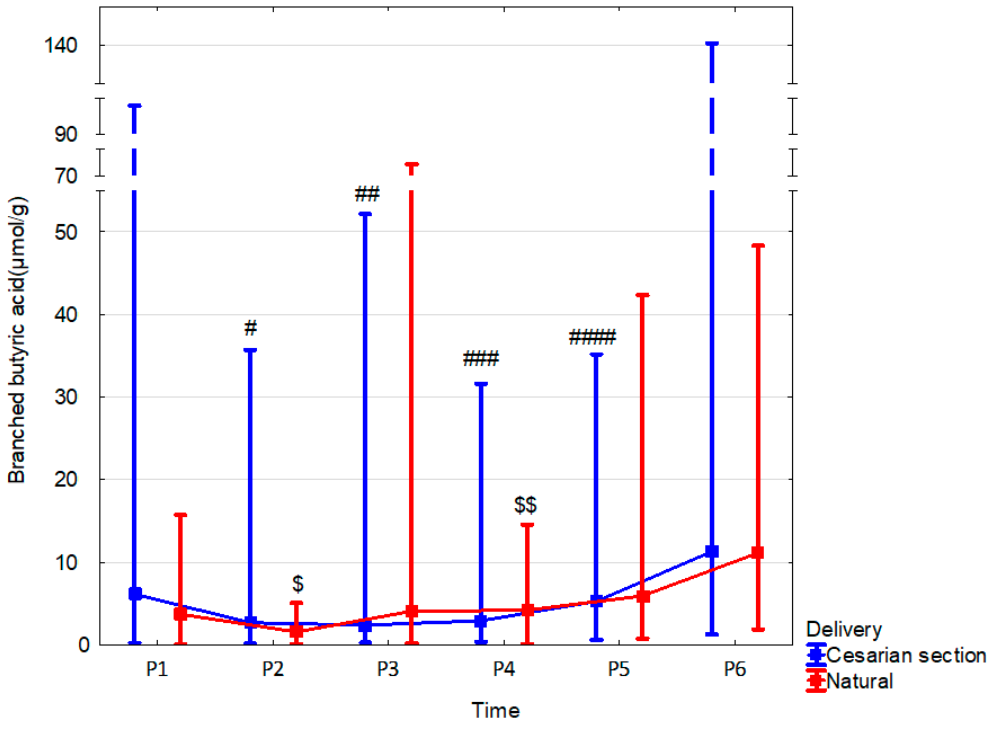

Figure S 3. Branched butyric acid concentrations (median) in children over time with delivery type: caesarean section (blue line) or vaginal birth (red line).

Legend: Error bars represent range. Wilcoxon paired tests regarding time, # (Caesarean section), $ (Vaginal delivery): #,$ p < 0.05 P2 vs P5 and P6; ##: p<0.05 P3 vs P6; $$,### p<0.05 P4 vs P6; #### p<0.05 P5 vs P6.

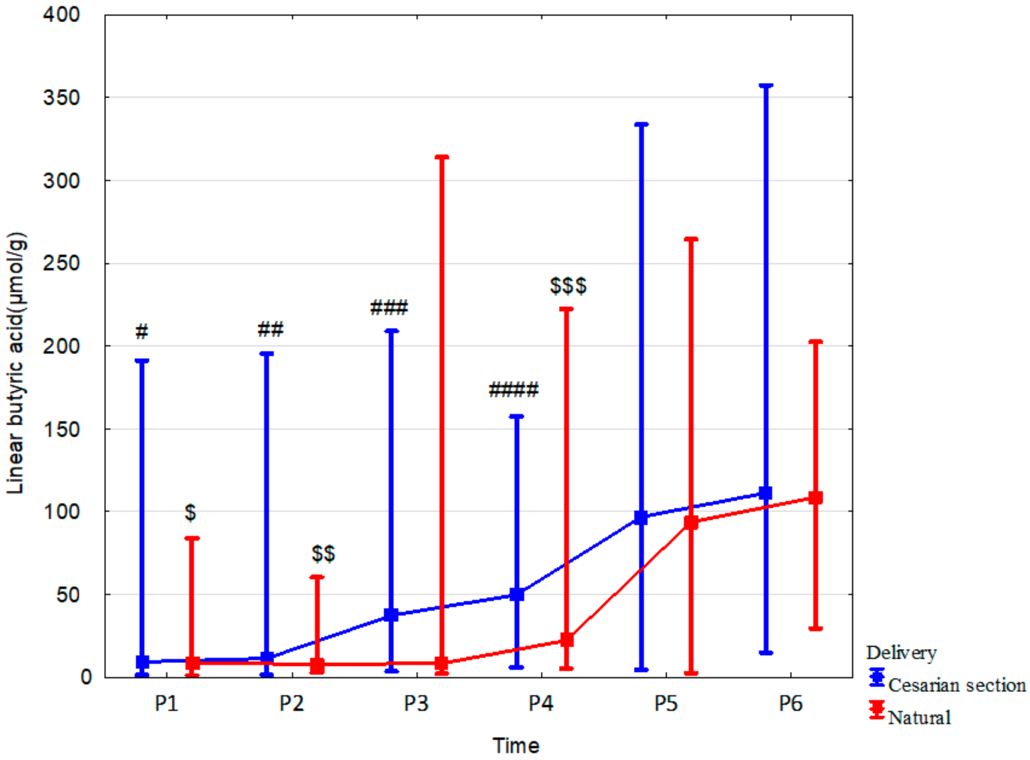

Figure S 4. Linear butyric acid concentrations (median) in children over time with delivery type: caesarean section (blue line) or vaginal birth (red line).

Legend: Error bars represent range. Wilcoxon paired tests regarding time, # (Caesarean section), $ (Vaginal delivery): #,$ $p < 0.05$ P1 vs P3,P5 and P6; ##,$$: $p < 0.05$ P2 vs P4,P5 and P6; ### $p<0.05$ P3 vs P4,P5 and P6; ####,$$$ $p<0.05$ P4 vs P5 and P6.

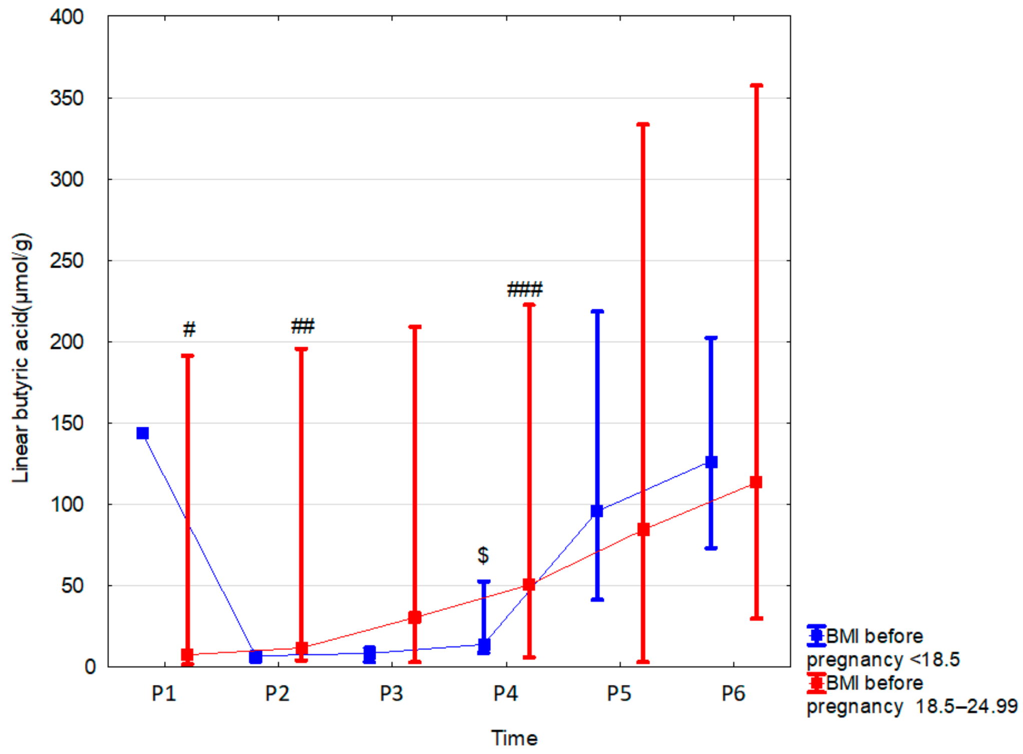

Figure S 5. Linear butyric acid (median) in children over time with effects of BMI before pregnancy: BMI before pregnancy 18.5-24.99 (red line) or BMI before pregnancy <18.5 (blue line).

Legend: Error bars represent range. Wilcoxon paired tests regarding time, # (BMI before pregnancy 18.5-24.99), $ (BMI before pregnancy <18.5): # p < 0.05 P1 vs P5; ##: p<0.05 P2 vs P5 and P6; ###,$ p<0.05 P4 vs P5 and P6.

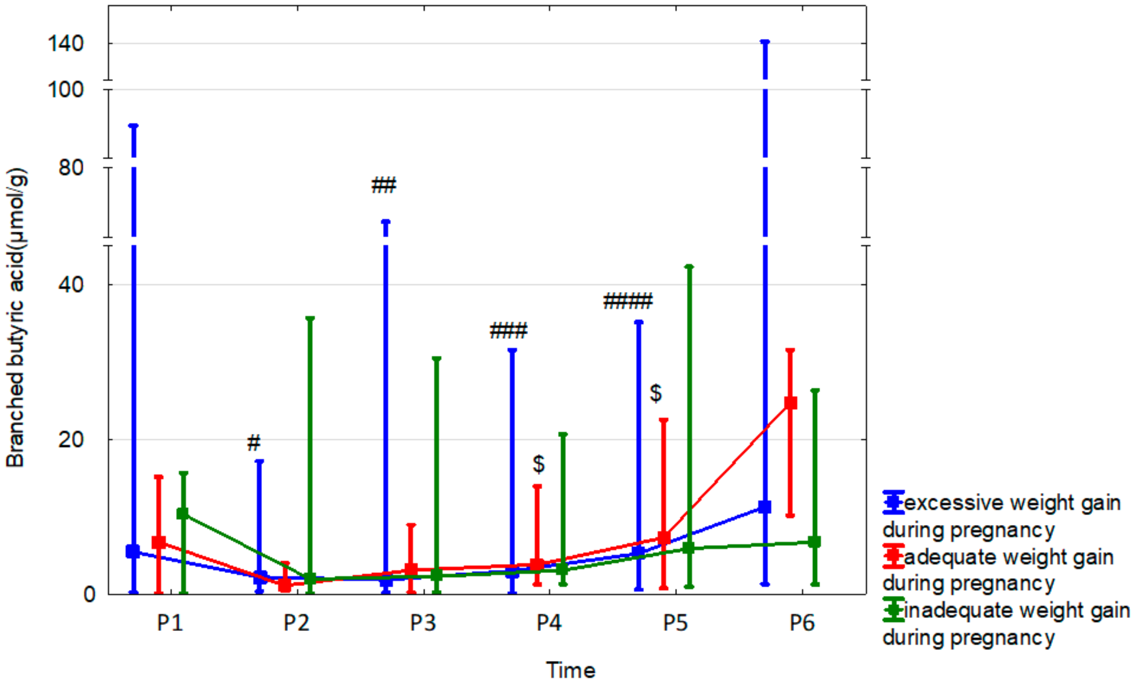

Figure S 6. Branched butyric acid (median) in children over time with effects of weight gain during pregnancy Excessive (blue line), Adequate (red line) or inadequate(green line).

Legend: Error bars represent range. Wilcoxon paired tests regarding time, # (excessive), $ (adequate), % (inadequate): # p<0.05 P2 vs P6; ## p<0.05 P3 vs P6; ###,$ p<0.05 P4 vs P6; ####,$$ p<0.05 P5 vs P6.

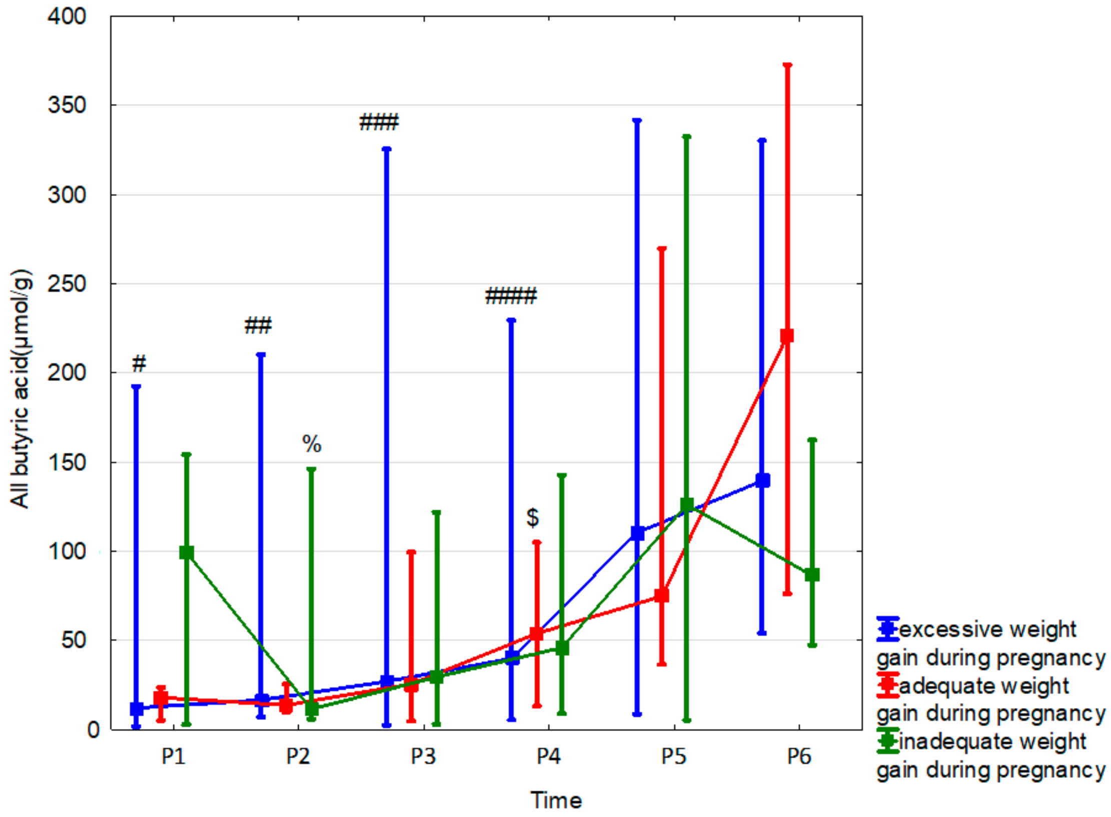

Figure S 7. All butyric acid (median) in children over time with effects of weight gain during pregnancy Excessive (blue line), Adequate (red line) or Inadequate (green line).

Legend: Error bars represent range. Wilcoxon paired tests regarding time, # (excessive), $ (adequate), % (inadequate): # p<0.05 P1 vs P2,P3 and P6; ##,% p<0.05 P2 vs P4,P5 and P6; ### p<0.05 P3 vs P5; ####,$ p<0.05 P4 vs P5 and P6.
